# Chitin and chitosan remodeling defines vegetative development and *Trichoderma* biocontrol

**DOI:** 10.1371/journal.ppat.1008320

**Published:** 2020-02-20

**Authors:** Lisa Kappel, Martin Münsterkötter, György Sipos, Carolina Escobar Rodriguez, Sabine Gruber

**Affiliations:** 1 Institute of Microbiology, University of Innsbruck, Innsbruck, Vienna, Austria; 2 Department of Functional Genomics and Bioinformatics, University of Sopron, Sopron, Hungary; University of Melbourne, AUSTRALIA

## Abstract

Fungal parasitism depends on the ability to invade host organisms and mandates adaptive cell wall remodeling to avoid detection and defense reactions by the host. All plant and human pathogens share invasive strategies, which aid to escape the chitin-triggered and chitin-targeted host immune system. Here we describe the full spectrum of the chitin/chitosan-modifying enzymes in the mycoparasite *Trichoderma atroviride* with a central role in cell wall remodeling. Rapid adaption to a variety of growth conditions, environmental stresses and host defense mechanisms such as oxidative stress depend on the concerted interplay of these enzymes and, ultimately, are necessary for the success of the mycoparasitic attack. To our knowledge, we provide the first in class description of chitin and associated glycopolymer synthesis in a mycoparasite and demonstrate that they are essential for biocontrol. Eight chitin synthases, six chitin deacetylases, additional chitinolytic enzymes, including six chitosanases, transglycosylases as well as accessory proteins are involved in this intricately regulated process. Systematic and biochemical classification, phenotypic characterization and mycoparasitic confrontation assays emphasize the importance of chitin and chitosan assembly in vegetative development and biocontrol in *T*. *atroviride*. Our findings critically contribute to understanding the molecular mechanism of chitin synthesis in filamentous fungi and mycoparasites with the overarching goal to selectively exploit the discovered biocontrol strategies.

## Author summary

Fungal pathogens pose an emerging threat in crop production and thus human health. *Trichoderma atroviride* is considered a potential biocontrol agent against a broad spectrum of phytopathogens. Cell wall chitin was identified as promising target to combat fungal diseases. Here we uncovered the regulation of chitin and chitosan synthesis and their contribution to dynamic cell wall remodeling as protective components in self-defense reactions during the mycoparasitic attack of *Trichoderma*. The systematic evaluation of the newly identified chitin-modifying enzymes confirmed their concerted interplay and their essential contribution to a successful mycoparasitic invasion. These findings provide further valuable, more specific information on targeting critical factors in the fungal cell wall adaptation process for therapeutic purposes as well as improved biocontrol applications.

## Introduction

Plant diseases are a widely acknowledged problem in industrial farming and often associated with a substantial loss of harvest. Nowadays combating plant pathogenic fungi mainly includes use of (petrochemical) fungicides. Such treatments however pose a significant environmental burden and are implicated in causing serious health problems [1]. Additional challenges, such as global warming with a rise in temperature leading to increased humidity or draught, can directly and/or indirectly promote the pathogen burden and pesticide resistance [2, 3]. This clearly mandates new techniques to control and avoid food and crop spoilage.

One of the most promising green alternatives to pesticides in agriculture are mycoparasites such as *Trichoderma* spp., which have successfully been used as a biocontrol agent since several decades now [4–8]. Mycoparasitic *Trichoderma* species attack and parasitize different plant pathogens, such as *Rhizoctonia spp*., *Phythium spp*., *Botrytis cinerea* and *Fusarium spp*. [6]. Notably, a comparative transcriptome study highlighted mycoparasitism as the ancestral life style of *Trichoderma* [9] making it the ideal candidate to investigate the underlying principles of mycoparasitism.

Even though sensing of the host as well as degradation of its cell wall (by e.g. chitinases, glucanases, proteases) have been extensively studied [10–20], critical aspects concerning *Trichoderma* cell wall remodeling during vegetative growth and invasion still remain elusive. It has been suggested, that the plasticity of *Trichoderma* cell walls might be fundamental for their mycoparasitic abilities [21]. Particularly chitin, and in some cases also its deacetylated form chitosan, are essential components in the cell wall of most fungi, and it seems puzzling that little to no data are available, on how the synthesis of these polymers contributes to mycoparasitism, as this process might be critical in recognition, penetration and lysis of a host. So far, it has only been known that the response of the host to mycoparasitism is accompanied by hyphal tip growth arrest and apical thickening [22], as well as a thickening of the cell wall around the infection structure of parasites, which coincides with up-regulation of chitin synthases and chitinases in some hosts [23].

Although chitin is only a comparably minor component of the fungal cell wall [24, 25] its strong micro-fibrils are most relevant for structural integrity [26]. In fact, chitin and chitosan seem to be important in protecting fungi against environmental stress factors, whether this might be temperature, osmotic stress or hostile enzymes [27]. Whereas yeasts contain only 0.5% to 5% of chitin, filamentous ascomycete cell walls contain up to 10–15% chitin fibrils [28, 29], consequently demanding a higher number of chitin synthases and chitinases in these fungi.

Much attention has been drawn to fungal chitin synthases, which are most extensively studied in *Saccharomyces cerevisiae*. However, comparison to filamentous fungi demands caution as *S*. *cerevisiae* comprises ‘only’ 3 synthases (ScChs1-3), with ScChs3 accounting for the bulk chitin synthesis *in vivo* [30–34].

Fungal chitin synthases are grouped into three divisions [35] and seven classes. Specific to filamentous fungi are only the classes III and V to VII. Class I-III belong to division 1 and are very simple in structure containing only the conserved chitin synthase domain. Class IV, V and VII belong to division 2 [36], and class V and VII contain an additional N-terminal myosin motor domain (MMD), which facilitates secretion [37, 38]. The class VI enzymes are the unique members of division 3, which share the conserved catalytic domain but no other characteristics.

Upon synthesis, chitin is secreted into the periplasmic space of the fungal cell wall, where it can further be deacetylated by specific chitin deacetylases (CDAs). Chitin deacetylases catalyze the hydrolysis of acetamido groups of the chitin substrate UDP-N-acetylglucosamine (GlcNAc) and act in tandem with chitin synthases *in vivo* [27, 39, 40]. A certain amount of deacetylation of the nascent chitin chain seems to be important in filamentous fungi to prevent chitin of forming too crystalline structures [41]. If more than 50% of the GlcNAc residues are deacetylated at position 2 [42] the heteropolymer is referred to as chitosan, which is positively charged in weak acidic environment and therefore less recalcitrant than chitin. Thus, fungal cell walls typically consist of a mixture of chitin and chitosan whose relative proportions vary depending on the taxa [26, 39].

One of the most relevant activities of CDAs was reported in plant–pathogen interactions, as chitin deacetylation is essential for cell wall rigidity and for resistance against chitinolytic enzymes secreted by the host. Therefore, extracellular CDAs are secreted during plant penetration to modify and thereby mask the chitin in the cell wall of the parasite, e.g. in *Colletotrichum lindemuthianum*, *Magnaporthe oryzae*, which could be recognized otherwise by a plant resistance system [43, 44]. Thus, chitin to chitosan conversion by CDAs may represent a sophisticated strategy towards hostile chitinases that *Trichoderma* mycoparasites are confronted with. Consequently, cell wall polysaccharide remodeling influences the antifungal resistance towards the pathogenic hosts and ensures that the mycoparasite prevails. This concept might be further expanded to enzymes involved in transglycosylation of chitin to glucan. Glucan/chitin cross-linking enzymes such as the CRH1 and CRH2 (UTR2) (originally described in *S*. *cerevisiae* [45]), represent critical players in fungal cell wall assembly [46]. Moreover, fungal chitosanases, from glycoside hydrolase family 75 (GH75), are another group of enzymes that participate in the decomposition of chitinous carbohydrates [47–49] and might thus be equally important in host interaction, lysis and cell wall remodeling. While most fungi have only 1–2 GH75 proteins, the mycoparasitic *Trichoderma* spp. have on average five [9]. Neither their transcriptional regulation nor their enzymatic properties were studied in these mycoparasites so far.

As outlined, the fungal cell wall is the first and crucial frontier in communication with the environment and during the mycoparasitic attack. The metabolism of chitin, as a key component of the cell wall, is under complex regulation. How mycoparasitic *Trichoderma* spp. guard their cell wall against own or hostile hydrolytic enzymes [21] remains largely uncharacterized. During fungal-plant interactions such findings were already reported [50, 51], and it remains to be shown whether similar concepts can be expanded to fungal-fungal systems. The present study focused on characterizing the full complement of chitin synthases and chitin deacetylases in *Trichoderma atroviride* using phylogenetic and domain structure analyses. We show that differential expression of these chitin and chitosan metabolic enzymes at different growth stages and during mycoparasitism is critical for a successful life cycle and that each of these enzymes is necessary in this concerted process (an overview is illustrated in the discussion). Importantly, a set of six chitosanases is also involved in mycoparasitism. To the best of our knowledge such a holistic analysis of chitin/chitosan metabolism has so far not been carried out in a mycoparasitic filamentous fungus. Our results therefore provide important insights in the role chitin and chitosan play during vegetative growth and improve our understanding of the mycoparasitic capability of *Trichoderma* as biocontrol agent.

## Results

### Phylogenetic and structural classification of chitin synthases (*chs*) and -deacetylases (*cda*) in *Trichoderma atroviride*

**Fig 1:**
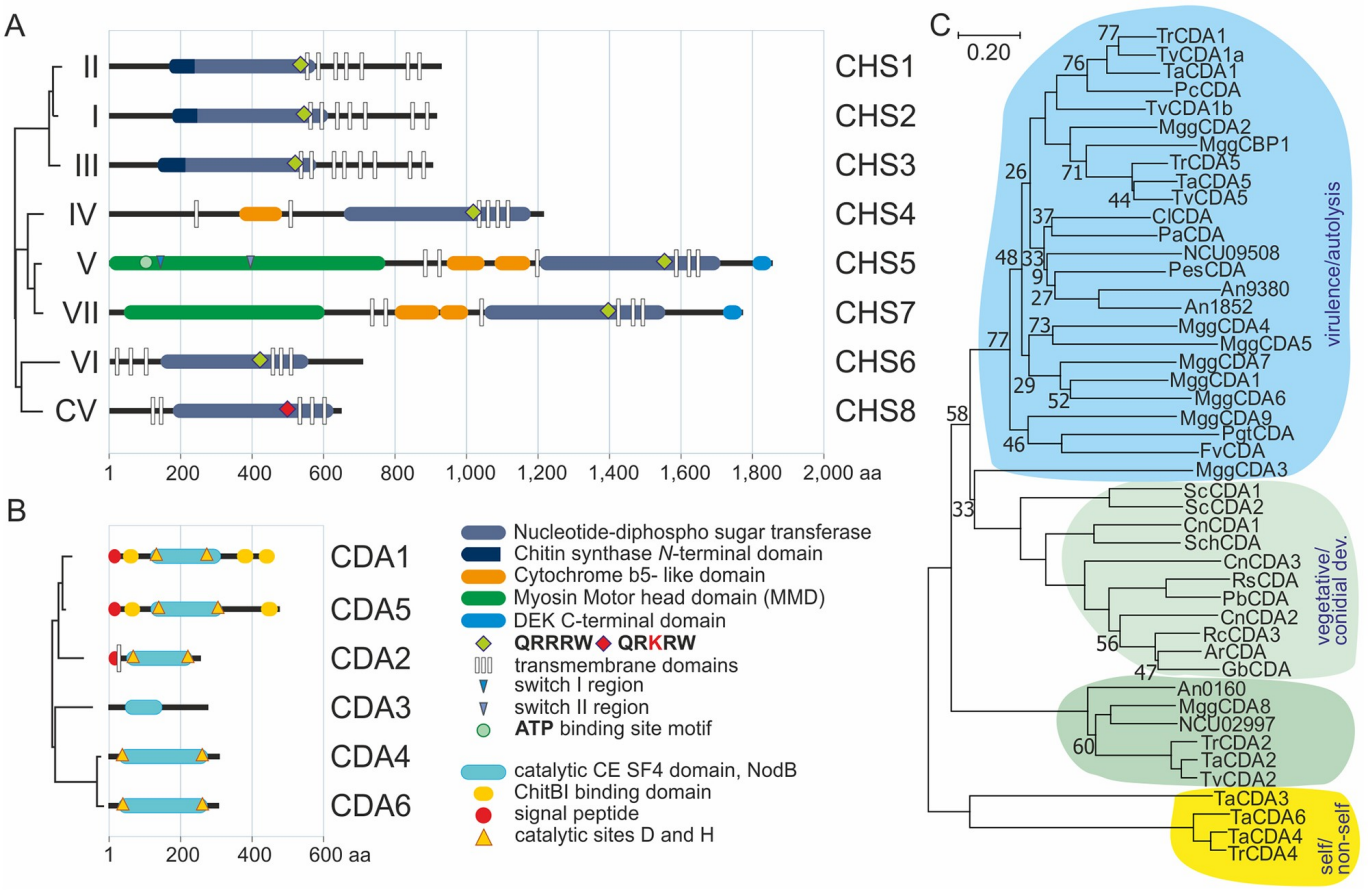
Phylogenetic and structural classification of chitin synthases (*chs*) and -deacetylases (*cda*) in *Trichoderma atroviride*. Structure/function prediction of the (A) eight chitin synthases (CHS1-8) and (B) six chitin deacetylases (CDA1-6). On the left side a condensed phylogenetic tree (dendrogram) is shown for both enzyme groups. (C) Phylogeny and functional relation of all six identified chitin deacetylases from *T*. *atroviride* in comparison to other organisms. Three functional groups are highlighted: light blue, virulence/autolysis related chitin deacetylases; light green/green, vegetative and conidial development-related transmembrane CDAs group I/ II; yellow, CDAs putatively involved in self-/nonself recognition. Bootstraps above 80 are not indicated. The bar marker indicates genetic distance. Gene name abbreviations are described in S1 Fig. A complete phylogeny of CHS1-8 in filamentous fungi is depicted in the S1 Fig.

Blast analysis using known chitin synthase sequences from *Aspergillus*, *Neurospora* and *Saccharomyces* identified eight genes in *Trichoderma atroviride* (IDs and references, S4 Table), of which seven can be directly assigned to the proposed classes I-VII [35, 52, 53]. The chitin synthases were designated CHS1-7 (Fig 1A and S1 Fig), with CHS1-3 and CHS6 representing chitin synthases with the most simple structure with only a chitin_synth_I (PF01644) or a chitin_synth_II motif (PF03142), respectively [35, 54]. CHS4 is the homolog of ScChs3p, which accounts for the bulk chitin synthesis in yeast [31, 54, 55], and contains a cytb5-like domain in addition to a chitin_synth_2 motif (Fig 1A). The related CHS5 and CHS7 additionally harbor a Dek domain at the C-terminus and a myosin motor head domain (MMD), but only CHS5 retained the functional ATP binding and switch I and II motives [38] (Fig 1A and S1 Fig).

The eighth chitin synthase (CHS8) falls out of the classification (with a QRKRW motif) and is present in a separate group together with the already identified chitin synthase FgCHS8 from *Fusarium graminearum*. FgCHS8 was shown to be important for virulence and fungal cell wall sensitivity to environmental stress [56]. This new fungal class of chitin synthases was also identified in a genome-wide analysis [53, 57, 58] where it was grouped into the proposed CV and ESV subclasses, which share high homology to chloroviral chitin synthases [59, 60]. Our analysis provides evidence, that they are most closely related to Class VI enzymes (S1 Fig).

In addition, blasting two known conserved chitin deacetylase motives from *S*. *cerevisiae* identified six chitin deacetylases in *T*. *atroviride* (Fig 1B). Subsequent phylogenetic analysis of the chitin deacetylases, using sequences of the saprotrophic and phytoparasitic ascomycetes and mucorales as comparators, revealed that CDA1 and CDA5 cluster together with deacetylases related to virulence [43, 61, 62]. They are most closely related to the only two *S*. *cerevisiae* chitin deacetylases (ScCda1 and ScCda2, [63, 64]; Fig 1B and 1C) due to the presence of a signal peptide and conserved type I chitin binding domains (ChitBI motifs/ CBM18) at their N- and C-termini, suggesting enhanced affinity to chitin polymers. However, ScCda1 and ScCda2 group to a separate clade with proteins involved in vegetative development and conidial maturation [63–65]. *Trichoderma* CDA2 is present next to this clade in a smaller separate group with chitin deacetylases that are cell wall associated and expressed during vegetative development, but lack the ChitBI motif (e.g. in *Magnaporthe oryzae* [43]). In contrast to the Zygomycete species *Amylomyces/Mucor rouxii* or *Cryptococcus neoformans* [39, 65, 66] in *T*. *atroviride* only CDA2 possesses also signature characteristics of membrane proteins (L4 –Y23; TMHMM server 2.0; http://www.cbs.dtu.dk/services/TMHMM/ [67]). *Trichoderma* CDA4 and CDA6 are highly homologous (76% identity/83% similarity) and only TaCDA3 and TrCDA4 were also found in addition in this fourth separate branch (Fig 1C). CDA3 diverges the most from all other CDAs mainly because it harbors a very degenerate chitin deacetylase domain that even lacks the conserved, catalytic aspartic acid and histidine residues (Fig 1B).

### The genomic environment of *chs* and *cda* reflects their specific roles

**Fig 2:**
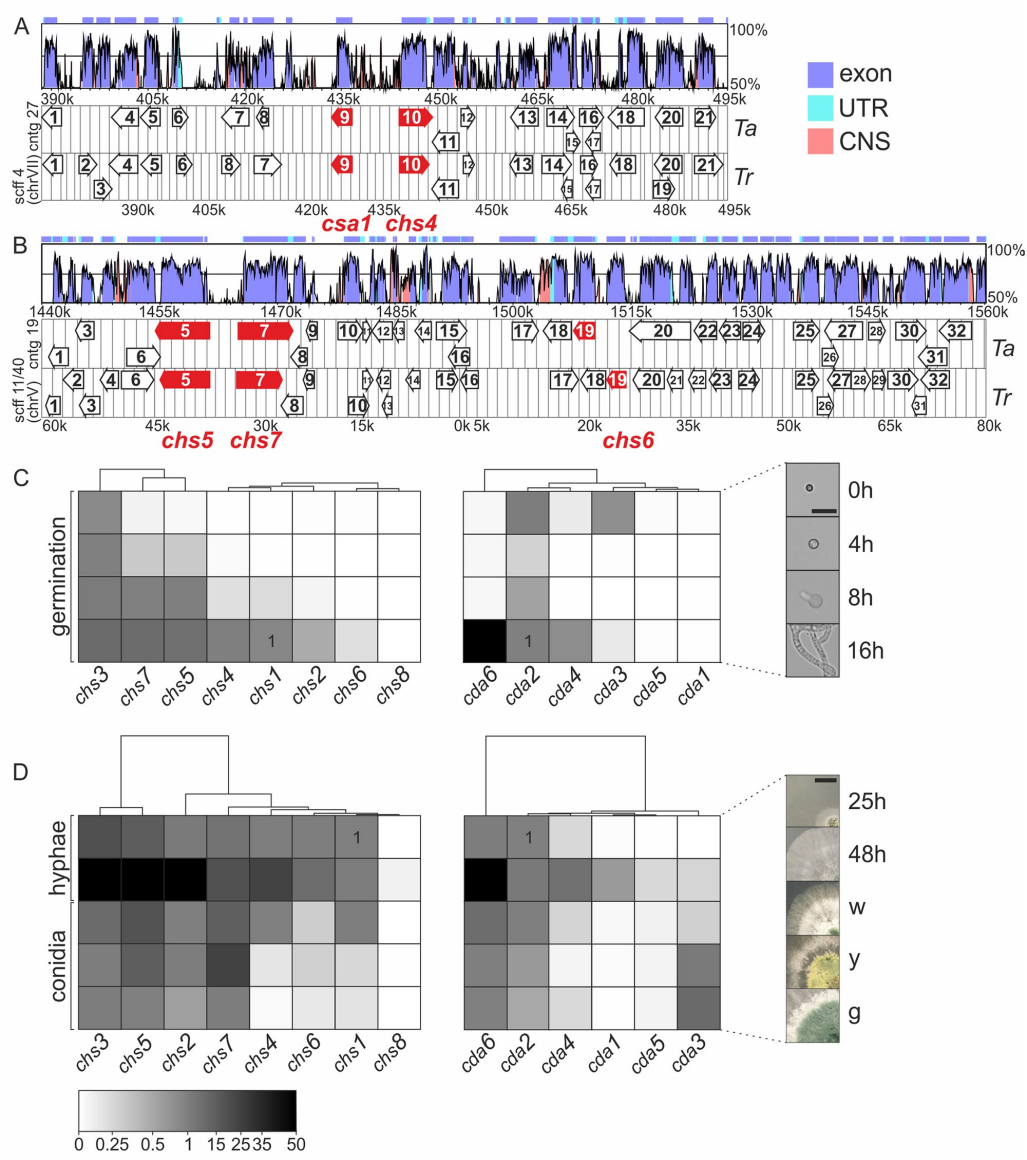
Genomic environment of *chs* and *cda* reflects their specific roles. (A, B) A cluster analysis was performed according to the chromosomal location of chitin synthase genes and their conservation among *T*. *atroviride* (Ta) and *T*. *reesei* (Tr) annotated at the JGI database for *Trichoderma atroviride* v2.0 (https://genome.jgi.doe.gov/Triat2/Triat2.home.html) and *Trichoderma reesei* v2.0 (https://genome.jgi.doe.gov/Trire2/Trire2.home.html). Conservation of exons (lilac), UTRs (untranslated regions, turquoise) and CNS (conserved non-coding sequences, red) is shown in the above panel of each diagram. Gene location on the chromosomes (*T*. *reesei*, [146]) and contigs (*T*. *atroviride*) is indicated in k = 1,000 nucleotides. Gene (white arrows) size and position are to scale and numbers correspond to gene description in S1 and S2 Tables. Chitin synthase genes and the activator are highlighted in red. (A) Gene cluster of chitin synthase (*chs4)* and the putative chitin synthase activator (*csa1*). (B) *chs5-chs7-chs6* gene cluster. (C-D) Transcript levels of chitin synthase (left panel) and chitin deacetylase genes (right panel) during vegetative growth and conidiation. Transcript levels of chitin synthase genes (*chs1-8*) were related to *chs1* and chitin deacetylase genes (*cda1-6*) to *cda2* expression at 16 h of germination for (C) and 24 h of growth on PDA for (D). Normalized expression of *chs1* and *cda2* corresponds to ‘1’ as indicated (see also S2 Fig). (C) Expression analysis during germination after 0 h, 4 h (isotropic growth), 8 h (formation of germination tube) and 16 h (first vegetative hyphae). Pictures of corresponding germination stages are shown on the right-hand side; scale bar = 20μm. (D) Expression analysis during hyphal development from mycelium grown on PDA for 24 h and 48 h and asexual development at different maturation stages with (w) white/ nascent, (y) yellow/immature and (g) green/mature conidia; scale bar = 1 cm.

Identifying conserved gene clusters is a useful bioinformatics approach to get first indications on putative functionalities, since it can help identifying regulators and co-factors for a certain pathway [68–70]. Hence, we performed a synteny analysis of chitin synthase and deacetylase genes in *T*. *atroviride*. A comparison of the genetic neighborhood with the saprophyte *T*. *reesei* provided further insights into the conservation of the clusters in related *Trichoderma* species with different life styles. We found that the organization of the *chs4* gene cluster identified in *Trichoderma* spp. strongly resembles that of the different *Aspergillus* species that were analyzed by Pacheco-Arjona et al. [69], (Fig 2A). *chs4* shows a chromosomal head-to-head arrangement with *csa1* (encoding the putative activator of class IV chitin synthases [29]), a gene organization typical for fungal secondary metabolism gene clusters or a coordinated transcriptional regulation of functionally related genes. *chs2* and *chs3* were also found on the same contig in *T*. *atroviride* and the same chromosome (VI) in *T*. *reesei*, but they are about 500 kb apart, so that co-regulation or shared regulatory elements are unlikely (*T*. *atroviride*: *chs2*: (contig_29) 659,292–663,807 *chs3*: (contig29) 1,153,752–1,157,194). While chitin synthases are involved in building up the cell wall, glycoside hydrolases, such as glucanases and chitinases, act antagonistically, by degrading the cell wall to allow cell growth. Indeed, a glucanase was identified upstream of *chs4* directly after *csa1*. Moreover, also the gene encoding NAG1 (a secreted *N*-acetylglucosaminidase) that is responsible for mobilization of GlcNAc from chitobiose [71] is located only 55 kb upstream from *chs4* in *Trichoderma* spp. Interestingly, the chitin deacetylase encoding gene *cda5*, was found in close vicinity to *nag2* (the second, but membrane bound *N*-acetylglucosaminidase) [71] on another scaffold in *T*. *atroviride* (contig 23: *cda5*: 1,914,660–1,916,233; *nag2*: 1,943,411–1,945,301) and two genes coding for chitosanases (CHO2 and CHO4) are located next to it but are both missing in *T*. *reesei*. A closer investigation of the genomic neighborhood of *chs5* showed that it most probably shares a bi-directional promoter with *chs7* since they are in a head-to-head arrangement, which was observed also in other fungi (Fig 2B and [38]). Furthermore, a serine/threonine kinase gene is found in close proximity of *chs5*, and a histidine kinase and -phosphatase down- and upstream, respectively, which might play crucial roles in activation of the myosin head of the chitin synthases as proposed by Pacheco-Arjona et al., [69]. *chs6* is located only 55 kb apart from *chs7* and contains additional regulatory and accessory genes in close vicinity (Fig 2B). *chs8* is located in a head to tail arrangement with *cda1* and a UDP-N-acetylglucosamine 6-dehydrogenase gene (UNGD) is located further upstream, which might also imply co-regulation, even though via separate promoters (Kappel and Gruber, manuscript in preparation). A similar arrangement was found in all fungi harboring this specific kind of chitin (hyaluronan) synthase [57]. Therefore, the chromosomal arrangements hint at a close transcriptional connection and imply a tight post-translational regulation of degradation and build-up of the cell wall in *Trichoderma spp*. Further experiments with the additionally identified genes need to be carried out to verify the proposed putative functions from the cluster analysis.

Transcriptional analysis during hyphal development (Fig 2C and 2D) showed that the chitin synthases and—deacetylases can be grouped into those with low and very low expression such as *chs8*, *cda1*, *cda3* and *cda5*, intermediate expression (*chs1*, *chs4*, *chs6*, *chs7*, *cda2*, *cda4*) and high expression (*chs2*, *chs3*, *chs5*, *cda6*). During generation of asexual conidia (from early white to yellow and mature, green spores (Fig 2D) the expression of the chitin synthases decreased slowly and only *chs1*, *chs2*, *chs3*, *chs5* and *chs7* were still expressed at intermediate levels in mature/green conidia (Fig 2D). Transcription of *cda2* and *cda6* was found in early stages of conidial maturation, but decreased also during late maturation. Interestingly, *cda3* transcription, that was very weak during all stages of hyphal growth, increased strongly during conidial maturation and might therefore be important in spore formation. High expression of *chs5* and *chs7* throughout hyphal development, vegetative growth and also conidial maturation was found (Fig 2C and 2D). Interestingly, *cda1* and *chs8* were only weakly expressed during vegetative development and also *cda5* expression was nearly undetectable, indicating involvement in other possibly stress related processes. These results outline the importance of chitin synthases *chs3*, *chs5* and *chs7* and *cda2*, *cda6* during vegetative development and *cda3*, as a crucial player in conidial maturation.

### Differential expression of *chs* and *cda* upon environmental stresses

**Fig 3:**
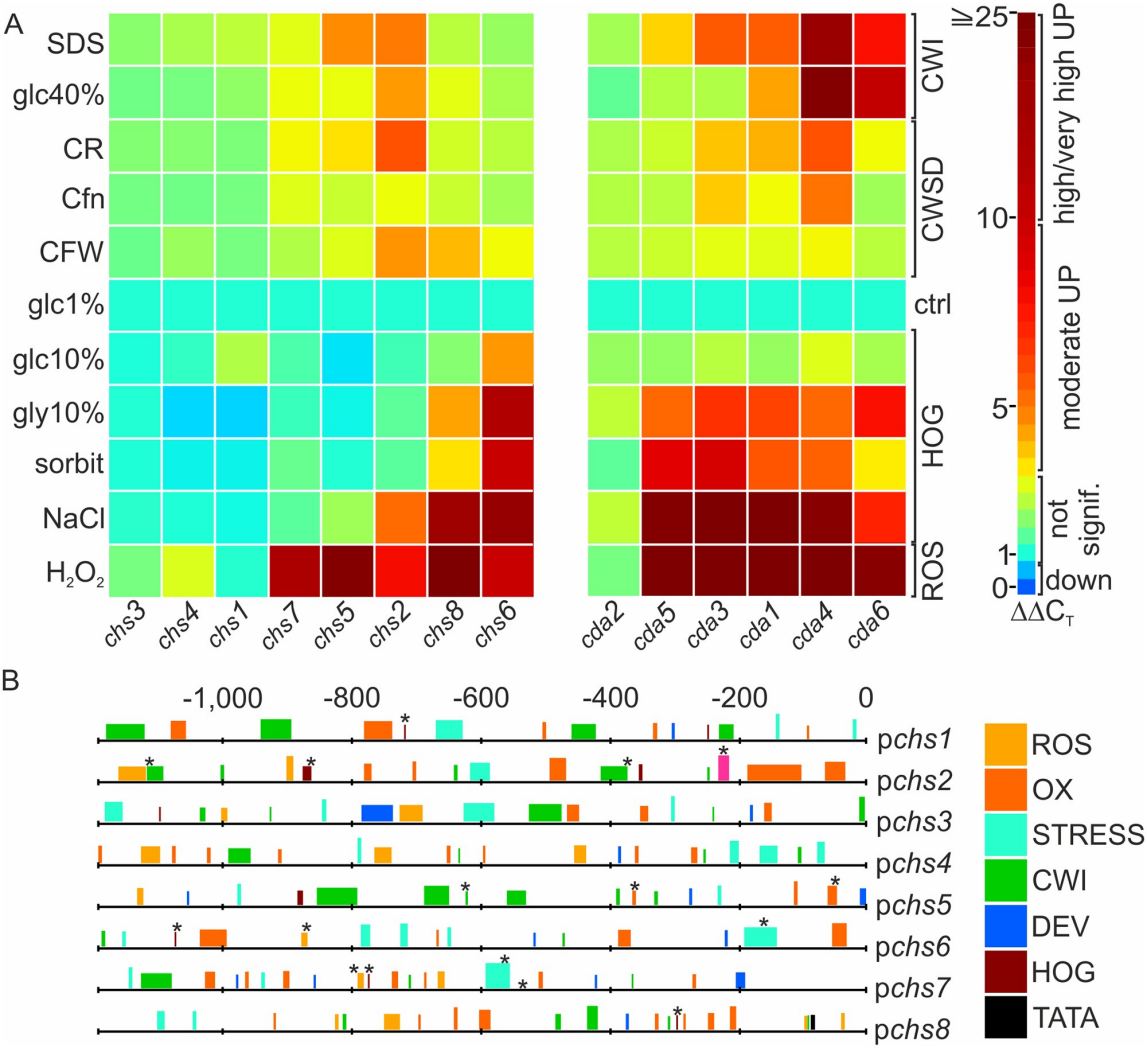
Differential expression of *chs* and *cda* upon environmental stresses. (A) Transcription analysis of chitin synthase (left panel) and chitin deacetylase (right panel) genes using various stressors. Comparison of growth under control conditions (glc1%, 1% glucose, ctrl) and with different stressors. On the left side the specific growth condition is indicated and on the right side a stress group is assigned: CWI (cell wall integrity disturbing stressors); CWSD (cell wall synthesis disturbing agents); HOG (high osmolarity glycerol) and ROS (reactive oxygen species). Detailed growth conditions are described in S3 Fig. The clustering results of transcription levels (values calculated by ΔΔC_T_ method) are represented in ‘jet’-style color mapping. (B) Graphical representation of the 1,200 bp upstream promoter regions of the eight chitin synthases and the identified stress element binding regions. Color code boxes indicate various stress groups whose height corresponds to the score level. CWI, HOG, ROS see (A); DEV, developmental regulation; OX, oxygen stress related; STRESS, general stress element; TATA, TATA-box or CAAT-box. Other detected and enriched motifs are marked with an asterisk. For more details on the promoter response elements see S3 Table.

Perturbation of fungal cell wall synthesis triggers a compensatory response to ensure cell wall integrity (CWI) with, among others, increased chitin synthesis. These stress responses are transduced via the CWI pathway. Perturbances that negatively affect cell wall composition and cell integrity such as osmotic shock and high salt levels (HOG), reactive oxygen species (ROS), or cell wall synthesis inhibitors (CWSD) are first sensed by integral membrane proteins and relayed to transcription factors. This leads to up- and downregulation of genes encoding cell wall synthesizing and modifying enzymes in a highly adaptive and effective manner [72, 73]. It has been shown that chitin synthesis and chitosan conversion is also influenced by external environmental stress factors in e.g. *Aspergillus* spp., *C*. *albicans* and *M*. *oryzae* [74–77]. To see if transcriptional regulation of chitin synthases and deacetylases also plays a critical role in *T*. *atroviride*, we investigated fold-changes of expression due to changed culture conditions simulating different environmental stresses. Interestingly, the expression of *chs1*, *chs3* and *chs4*, as well as *cda2*, which all were expressed throughout hyphal development, was not up-regulated upon any of the tested stress condition. Only *chs6* and *chs8* expression was increased up to 14-fold upon osmotic stress (Fig 3A). Remarkably, a hypothetical HSP88, family HSP110/HSP70 encoding gene, is located in a head to tail arrangement downstream of *chs6* (Fig 2B). Among the deacetylases *cda3*, *cda1* (up to 32-fold) and, to a lower extent, *cda4*, *cda5* and *cda6* (up to 21-fold) responded to HOG stress. A moderate response in transcription was observed towards cell wall disturbing agents, were especially *chs2*, but also *chs5* and *chs7* as well as *cda1*, *cda*3 and *cda*4 showed the strongest response with up to 6-fold increased transcript levels (Fig 3A). *chs2* levels also increased moderately (5-fold) when more harsh treatments such as 40% glucose and SDS were applied, which eventually resulted in hyphal lysis (S3 Fig). *cda4* (19-fold) and *cda6* (10-fold), but also *cda1* and *cda3* levels (5.5-fold) were increased under these conditions (Fig 3A). During the mycoparasitic attack fungal hosts use defense strategies such as generation of reactive oxygen species [78]. Interestingly, ROS created from incubation with H_2_O_2_ resulted in very strong upregulation of most chitin synthases (48-fold, exceptions *chs1*, *chs3*, *chs4*) and chitin deacetylases (145-fold, exception *cda2*). *In silico* analysis of all chitin synthase promoters using known motives for transcription factor binding sites from *S*. *cerevisiae* corroborated our results from the transcriptional approach. An enrichment of stress related elements in the promoter region of the strongest stress responders from the expression analysis *chs2*, *chs5*, *chs6*, *chs7* and *chs8*, in comparison to the other chitin synthases was confirmed (Fig 3B, S3 Table). By contrast, developmental elements were rather found in *chs3*, *chs5* and *chs7*. The occurrence of oxidative stress elements was especially high in *chs2* and *chs8*, whereas CWI-stress related elements were enriched in the MMD-domain *chs5* and *chs7* as well as in *chs2* and the other two division I chitin synthases. Thus, our findings provide evidence that especially *chs2* and *chs6* as well as chitin deacetylase transcription, with the notable exception of *cda2*, is highly adaptive and that *T*. *atroviride* is well prepared for and capable of fast reaction to a changing environment and defense reactions by the host during the mycoparasitic attack.

### Critical roles of *chs* and *cda* in growth and development are emphasized in single knockout mutants

To study the role of the genes identified, we generated corresponding knockout lines. We assumed that null mutants of non-redundant cell wall remodeling genes could repress growth or even be lethal given their importance for fungal development and survival. Nonetheless, we were able to generate single deletion strains of all chitin synthase and chitin deacetylase genes, except for *cda4*. The deletion of *cda4* proved difficult even after several rounds of transformation (ectopic integration detected > 20). Whether this is due to the gene being essential or if the gene is located at an inaccessible position on the chromosome remains elusive.

**Fig 4:**
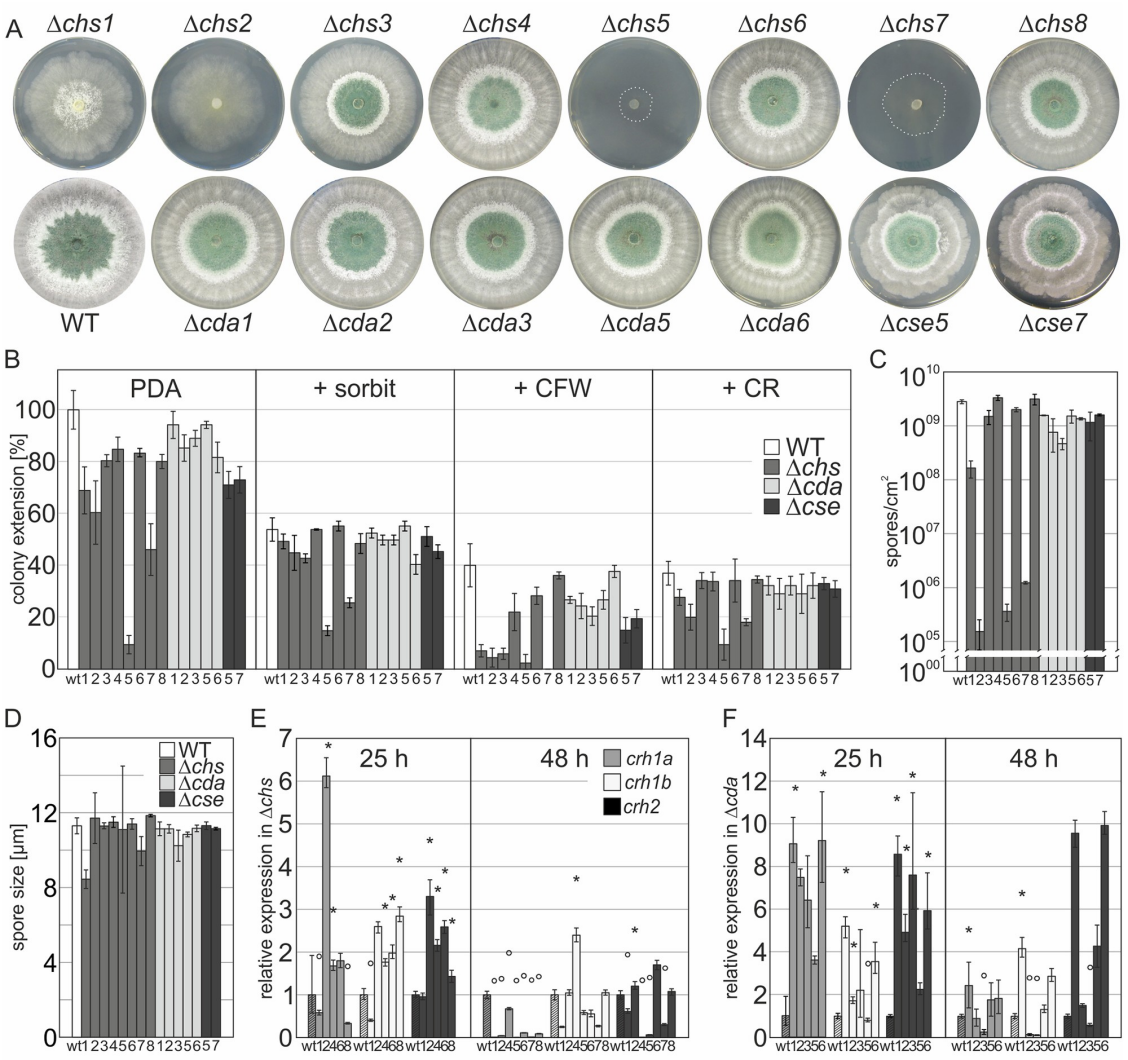
Critical roles of *chs* and *cda* in growth and development are emphasized in single knockout mutants. (A) Growth of generated deletion mutants and the *T*. *atroviride* wild type strain (WT) on PDA after 72 h. (B) Colony extension in percent of the parental strain (wt). Growth on PDA only and on plates containing 1.2 M sorbitol (+sorbit), 200 μg/ml calcofluor white (+CFW) or 150 μg/ml congo red (+CR) was compared. (C, D) Generation of asexual conidia by all deletion mutants, see also S4A Fig; (C) spores/cm^2^ and (D) average conidial size. (E-F) Compensatory transcription analysis of the transglycosylase genes *crh1a*, *crh1b* and *crh2* in the Δ*chs* (E) and Δ*cda* (F) strains compared to expression in the WT strain after 25 h and 48 h of growth on PDA (*tef1* used as housekeeping gene). Mean +/- SEM are shown and p>0.005 are indicated with *, upregulated; °, downregulated. (B-F) Data was generated from at least two independent experiments.

Colony extensions of all deletion strains and their susceptibility to stress were assayed on solid medium (Fig 4A and 4B). Significant differences were observed after 3 to 7 days of growth between the WT and mutant strains, suggesting half of the chitin synthases are indispensable for optimal growth under the tested conditions. Especially the MMD-chitin synthase mutants Δ*chs5* and Δ*chs7* were severely compromised in development with irregular colony appearance and thin mycelial network formation. In addition, little or no conidial production with irregular size was observed (Fig 4C and 4D). These severe defects in group V and VII CHS have also been reported for other fungi and they play a crucial role in virulence in plant pathogens [38, 79]. Δ*chs1* and Δ*chs2* show a considerably reduced growth rate on PDA. But unlike *chs5* and *chs7* KO strains, division I synthase mutants form a dense hyphal matrix suggesting aberrant branching frequency or hyphal fusion in this area [80]. *Δchs1*, *Δchs2*, had strongly decreased amounts of spores and *Δchs1*, *Δchs2*, *Δchs5* and *Δchs7* produced mainly whitish to yellow spores, even when grown with 1.2 M sorbitol as osmotic stabilizer (S4B Fig), indicating a critical role of chitin biosynthesis in conidial maturation (Fig 4C and 4D).

When strains were exposed to the cell wall intercalating dyes Calcofluor white (CFW) and Congo red (CR) to determine changes in the cell wall composition, the parental strain already showed reduced growth rates with about 60% inhibition. Interestingly, unlike other mutant strains tested in our experiments, group V and VII CHS cannot compensate for the cell wall intercalator (~95% inhibition; Fig 4B). Furthermore, all three division I chitin synthase knockout strains have severe growth defects (85–90% inhibition) on the cell wall dyes (Fig 4B). These findings corroborate our hypotheses drawn from the transcriptional profiles that these *chs* play fundamental functional roles in germination and vegetative development (Fig 2C and 2D). Interestingly though, the slower hyphal elongation of Δ*chs1*, *Δchs2*, *Δchs4* and *Δchs6* was almost completely recovered in the presence of 1.2 M sorbitol, but only slightly in the *Δchs5 and Δchs7* strains. Note that the parental strain was also very sensitive to sorbitol on solid medium (~ 50% reduction) whereas in liquid medium this effect is negligible (S3 Fig, [81]).

In *S*. *cerevisiae* ScChs3 chitin synthase activity is influenced by activators such as the ScChs4/ScSkt5 and other proteins such as ScChs5, ScChs6 and ScChs7 [29, 82, 83]. ScChs5/ScChs6 are involved in the polarized transport of ScChs3 in specialized vesicles (the so called exomer complex [84–86]), where ScChs5 seems to serve as the major adaptor for ScChs3 and ScChs7 controls export of ScChs3p from the endoplasmic reticulum (ER). Among these (additional) regulators, the Chs7 chaperone seems to be critical to mediate ScChs3 (TaCHS4) activity, which has been confirmed recently in *N*. *crassa* and *F*. *oxysporum* [87, 88]. We therefore identified the homologs of the two most important proteins in *T*. *atroviride* (pID 179314, 158601) and designated them Chitin Synthase Export chaperone, CSE7 (ScChs7/NcCSE7) as suggested by Rico-Ramirez et al., [87], and CSE5 (ScChs5) Chitin Synthase Exomer adaptor. The deletion of each of the genes affected growth on PDA (~ 30% reduced) and showed an obvious defect when grown on CFW (50–60% reduced compared to WT). The Δ*cse5* and Δ*cse7* built a highly layered, laminar mycelium with abundantly branched mycelial mats stacked upon each other leading to a very irregular colony diameter (Fig 4).

Among the chitin deacetylase knockouts, Δ*cda*2 and Δ*cda*3 were suppressed at an early stage of growth (0–36 h, S4A Fig), and the radial colony had a dense network formation phenotype similar to single Δ*chs1/* Δ*chs2*. Both strains also produced significantly reduced amounts of spores (Fig 4C and 4D). Δ*cda6* showed a concrete green ring at the colony center with crystalline spores (Fig 4 and S4A Fig), and minor effects, when treated with CFW or CR, suggesting minor changes in polysaccharide architecture. All complemented strains (with the WT gene reintroduced at a random site or gene replacement) showed the WT phenotype and conidiation pattern (S5 Fig).

In order to find additional evidence that the lack of chitin synthases leads to divergence of cell wall structuring and therefore affects cell wall remodeling, polysaccharide assembly and linkage, we were interested in the three most important chitin-glucan transglycosylases in *Trichoderma atroviride*. In the course of our work we identified *crh1a*, *crh1b* and *crh2*, that are highly homologous to ScCrh1 and ScCrh2, which catalyze the cross-linking of chitin and β-1,6-glucan [45, 46]. In the chitin synthase mutants they were significantly down-regulated after 48 h of development, especially in Δ*chs5* and Δ*chs7* (Fig 4E). During this later stage of maturation, hyphal fusion normally produces a dense mycelial network, which induces high expression of transglycosylases. The significant down-regulation of *crh1a* in all investigated *Δchs* is an additional hint towards co-regulation and a strong evidence for altered cell wall morphology in *chs* mutants (Fig 4E). In contrast to *Δchs*, the transglycosylases were found strongly upregulated in the *cda* deletion strains, hinting at a compensatory action to decreased cell wall flexibility in these mutants [89, 90] (Fig 4F).

In conclusion, functional analysis of mutant strains strongly suggests that at least 5 chitin synthases and 3 chitin deacetylases are critical for vegetative development including conidial maturation. Our findings highlight the importance of chitin, and most probably chitosan, for cell wall integrity during asexual development and in a hostile environment.

### Single knockout mutants show severe morphological changes and aberrant chitin deposition in patches with reduced chitin levels

**Fig 5:**
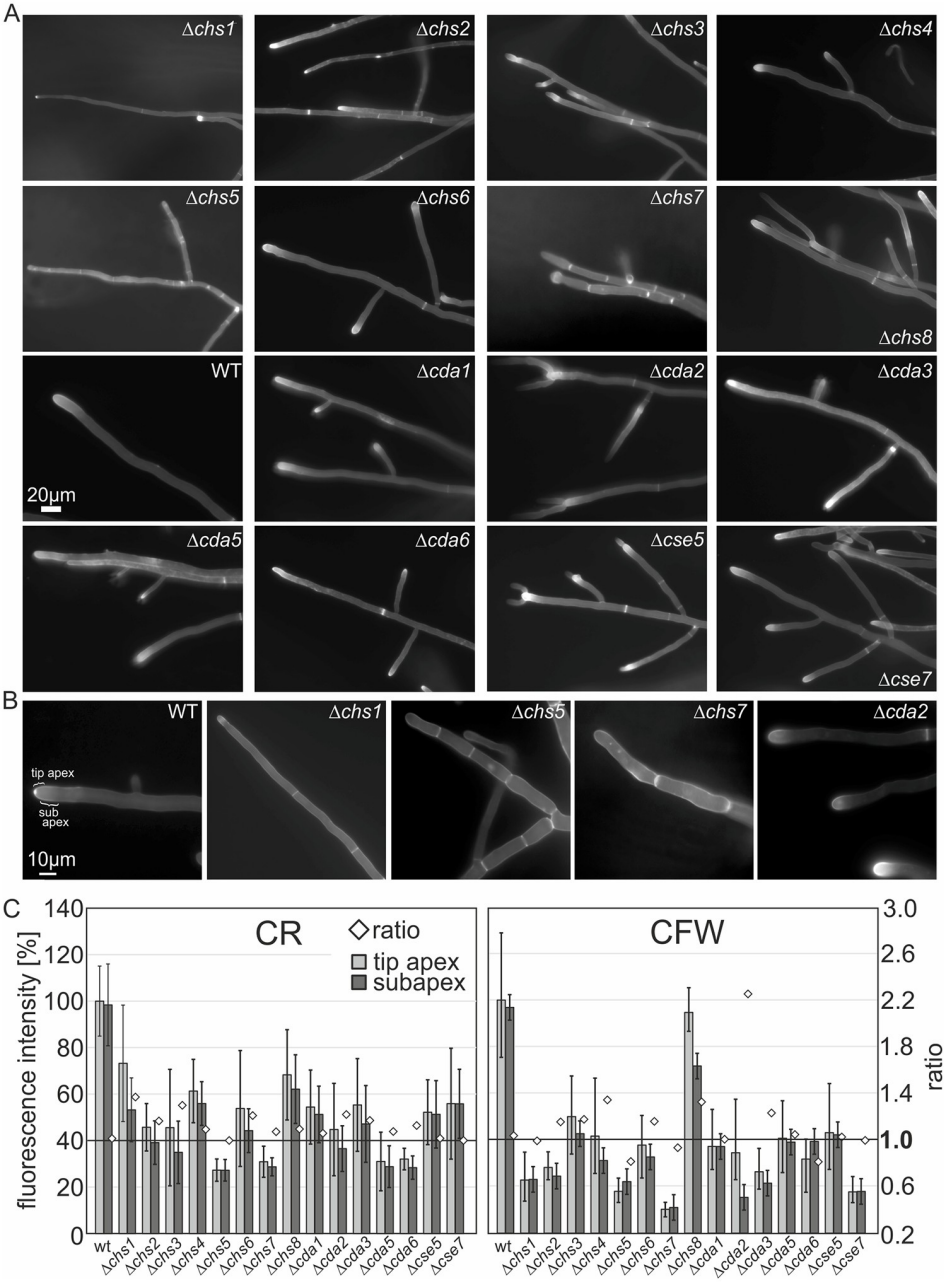
Single knockout mutants show severe morphological changes and aberrant chitin deposition in patches with reduced chitin levels. (A) Chitin deposition and tip morphology in leading hyphae of the peripheral zone of each colony was visualized by CFW staining in epifluorescence microcopy using the inverted agar method for live-cell imaging [91]; scale bar = 20μm. (B) CFW staining highlights aberrant chitin deposition and septal development in selected deletion mutants; scale bar = 10μm. (C) Altered cell wall chitin deposition in *chs* and *cda* deletion strains compared to the WT was investigated by semiquantitative fluorescence microscopy using CFW and CR as stains. A defined area of the tip apex and subapex (shown in B) of leading hyphae was analyzed by densitometry using the ImageJ software platform (http://rsb.info.nih.gov/ij/). Mean +/- SEM from n = 25–60 hyphae are shown.

Deletion of chitin synthases and—deacetylases affects cell wall composition and distribution of cell wall components (Fig 4; [27, 52, 65]) and as a result hyphal morphology is equally compromised. Microscopic analysis of mutant strain morphology using the inverted agar method [91] showed an aberrant hyphal development, with a weak and thin mycelial network and fewer branches in Δ*chs1* in comparison to the *T*. *atroviride* wild type. To identify growth characteristics that might be linked to aberrant chitin synthesis, epifluorescence microscopy was performed using the fluorochrome CFW, which stains β-1,4-linked polysaccharides such as chitin. In the wild type, CFW fluorescence was strongest at the septa and tips, where chitin is actively synthesized and weaker staining occurred at the lateral walls. Both, Δ*chs1* and Δ*chs2* had considerably thinner hyphae and branching started very late, and more distant from the tip than in the parental strain (Fig 5A and 5B, S4C Fig). In addition, Δ*chs2* showed increased CFW staining indicating higher deposition of chitin at the tip and lateral walls. Δ*chs4*, Δ*chs6* and Δ*chs8* did not show pronounced morphological aberrations, but only a slight increase in hyphal tip staining (Fig 5A). Phenotypic characterization of *T*. *atroviride* Δ*chs5* and Δ*chs7* revealed the most severe defects. They showed short lateral hyperbranching alternated with long, thin filamentous hyphae without branches (S4C Fig) and grew irregularly, with strongly inhibited hyphal elongation and chitin accumulated in patches at the lateral walls (Fig 5A and 5B). Remarkably, Δ*chs5*/ Δ*chs7* showed an elevated frequency in septation together with severe constrictions, possibly caused by a disproportionate distribution of chitin between the septa and the lateral walls. In addition, these mutants seemed inhibited in apical growth and often extensive isotropic tip swelling (increased sensitivity to CFW suggesting a cell wall weakening) was observed (Fig 5B).

Microscopic examination of all *cda* mutants showed pronounced staining of CFW at their apices in comparison to the lateral walls suggesting aberrant chitin accumulation, resulting in a brighter Spitzenkörper staining in all *cda* mutant strains. Quantification of the ratio of the relative dye fluorescence between tip apex and subapex with CFW and CR (Fig 5C), which more specifically binds to chitin than CFW, clearly showed that significantly less dye was incorporated into the cell walls of all deletion mutants. Only Δ*chs8* showed CR staining comparable to the WT in the hyphal tips but staining was also reduced in the cell wall in the sub apical zone (Fig 5C). This change in the cell wall deposition pattern of tip apex to sub apex was also observed in all of the three class I *chs* as well as *chs6* knockout and the Δ*cda*. The highest ratio (2.2) was observed in the *cda2* knockout strain for CFW (Fig 5B and 5C). Baker et al., also reported a strong accumulation of chitin and very low levels of chitosan in the chitin deacetylase deletion strains in *C*. *neoformans* [65].

Moreover, we adapted the biochemical assay for cell wall chitin determination described in this publication ([27, 65]; see Materials and methods) to get further insights in the impact on chitin synthesis in the cell wall of the *Trichoderma* mutants. The analysis of the vegetative hyphae of all mutants confirmed that a strong reduction of chitin in the cell wall was caused by the absence of most of the chitin synthases, *cse5* and *cda6*, with the lowest amounts in Δ*chs5* and Δ*chs7* (48 and 37%), while deletion of the other deacetylases, or *cse7* resulted only in a 84–93% (Table 1). Deletion of *chs2*, *chs6* and *cda5* did not impact chitin levels under the tested growth condition.

**Table 1:** Relative GlcNAc content in the cell wall of mutant strains compared to the wild type.

	GlcNAc [%]	
WT	**99.7**	+/- 6.1
Δ*chs1*	**62.6**	+/- 12.9
Δ*chs2*	**98.6**	+/- 7.4
Δ*chs3*	**63.7**	+/- 22.0
Δ*chs4*	**83.7**	+/- 14.6
Δ*chs5*	**47.9**	+/- 17.1
Δ*chs6*	**99.7**	+/- 14.3
Δ*chs7*	**32.4**	+/- 9.4
Δ*chs8*	**87.2**	+/- 8.6
Δ*cda1*	**93.1**	+/- 19.6
Δ*cda2*	**91.7**	+/- 21.4
Δ*cda3*	**86.8**	+/- 20.0
Δ*cda5*	**100.5**	+/- 13.6
Δ*cda6*	**66.4**	+/- 12.2
Δ*cse5*	**76.9**	+/- 9.4
Δ*cse7*	**83.7**	+/- 7. 7

n = 9 (three independent experiments with three technical replicates)

Microscopic analysis of Δ*cda2*, Δ*cda3* and Δ*cda6*, Δ*cse5* and Δ*cse7* showed increased branching with dichotomous branching indicative of a dysregulated apical dominance and also more lateral branches (Fig 5A). *Δcda5* revealed a loss of hyphal avoidance, and accumulation of lateral and cytoplasmic staining. In summary, in most of the Δ*chs* (predominately Δ*chs2/5/7*), and Δ*cda*, patches of bright fluorescence were observed at the lateral wall of hyphae. This altered distribution of cell wall components seems to interfere drastically with polarized growth and branching patterns. Our results indicate that aberrant cell wall synthesis activities caused by absence of nearly any of the synthesizing enzymes lead to reduced chitin levels and altered distribution of chitin and putatively other cell wall polymers in the mutant strains, which strongly affects their hyphal development.

### Mycoparasitism depends on coordinated expression of chitin synthases, -deacetylases and chitosanases

**Fig 6:**
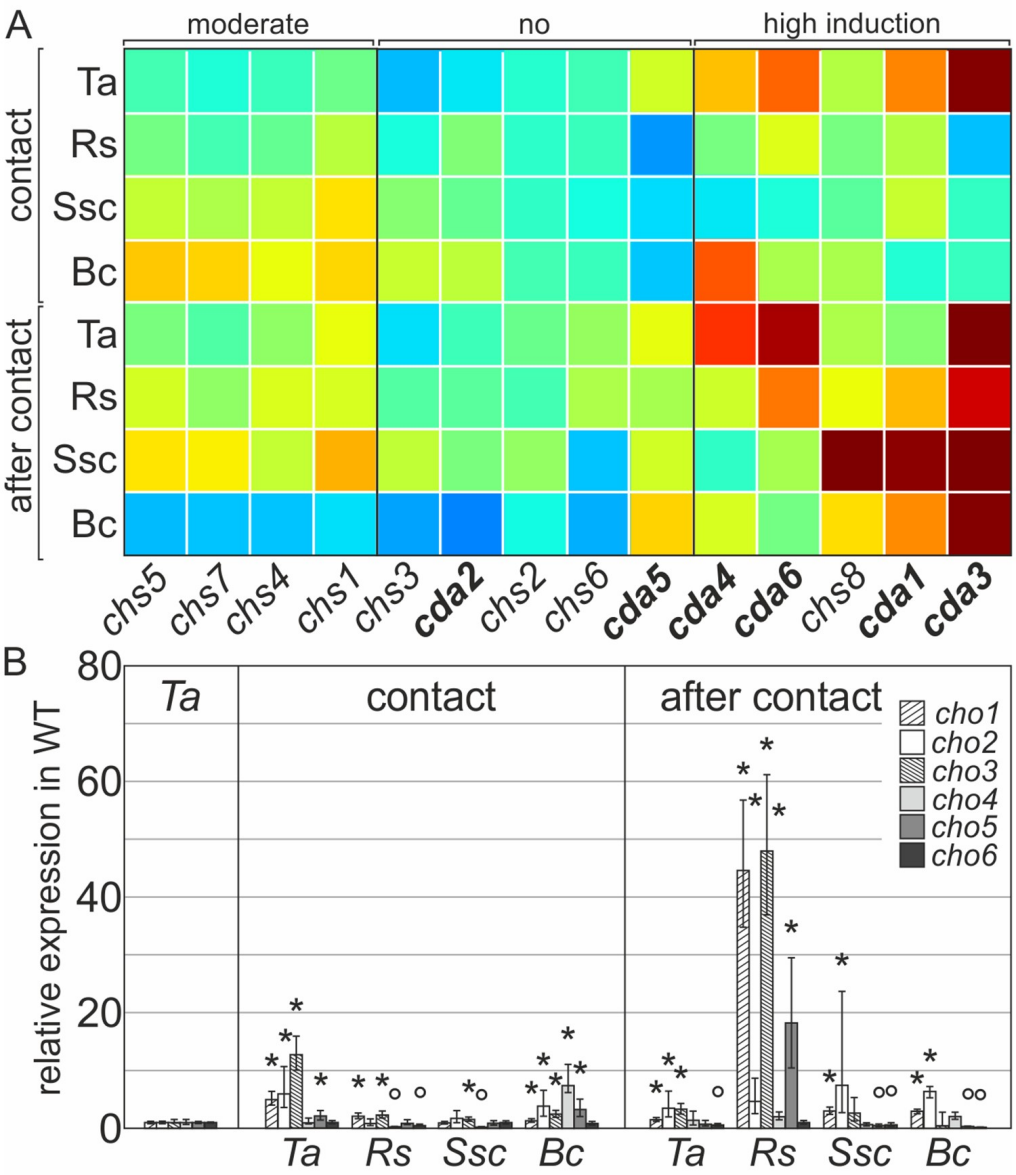
Mycoparasitism depends on coordinated expression of chitin synthases,–deacetylases and chitosanases. Differential transcription analysis of chitin synthases, chitin deacetylases and chitosanases during the mycoparasitic attack of *T*. *atroviride* against three different hosts. Expression of all target genes was analyzed using the ΔΔC_T_ method. *T*. *atroviride* against itself (Ta), *R*. *solani* (Rs), *S*. *sclerotiorum* (Ssc) or *B*. *cinerea* (Bc). The WT grown alone on a plate (control condition) was arbitrarily set to ‘1’. Samples were collected at contact and after contact (4 mm overgrowth). The gene *sar1* was used as the housekeeping gene. Data were generated from two independent experiments and three technical replicates. Mean +/- SEM and p>0.005 are indicated with *, upregulated; °, downregulated. (A) Transcription analysis in confrontation assays with fungal hosts, *chs1-8*, chitin synthase; *cda1-6*, chitin deacetylase. The clustering results are represented in ‘jet’-style color mapping as described in Fig 3A. (B) Relative expression of all identified chitosanase genes (*cho1*-*cho6*) during mycoparasitism.

Mycoparasitism by *Trichoderma* has been studied already extensively and many critical events have been elucidated but what remains still unknown is how exactly mycoparasitic species remodel their own cell walls to circumvent the defense mechanisms of phytopathogenic fungi that themselves comprise an aggressive plethora of chitinolytic enzymes during the invasive process. We therefore first tested the expression of chitin and chitosan metabolic enzymes, when *T*. *atroviride* was confronted with itself or the fungal hosts *S*. *sclerotiorum*, *B*. *cinerea* and *R*. *solani*. Expression analysis revealed exclusively high induction of *chs8* and *cda1* expression under mycoparasitic conditions during confrontation with all hosts (Fig 6A). The concerted activation of both enzymes is possibly due to their genomic arrangement in a gene cluster driven by a shared regulatory element (Kappel and Gruber, manuscript in preparation). Interestingly, *chs1*, *chs4*, *chs5* and *chs7* were also moderately upregulated during the mycoparasitic attack next to their critical role in vegetative growth. The upregulation of *chs5/ chs7* during mycoparasitism (Fig 6A) highlights the importance of the MMD containing synthases in any pathogenic event, either phyto- or mycoparasitic. Interestingly, *cda3*, *cda4* and *cda6* were induced in self-recognition of the fungus (control condition: *Ta)*, indicating a general function in cell wall remodeling (synthesis and repair), and primary growth. Transcription of the stress responders *chs2*/6 and the genes for vegetative development *chs3*/*cda2* was not significantly altered during mycoparasitism (Fig 6A).

As discussed, cell wall turnover, chitin crosslinking to glucan, as well as its conversion to chitosan might be critical for the antagonistic action of *Trichoderma* towards its prey. Equally important seems the role of chitosanases, which hydrolyze chitosan and chitooligosaccharides in host interaction, lysis and cell wall remodeling, an area currently of particular attention [47]. We identified 6 chitosanases in *T*. *atroviride*. CHO1 and CHO2 show high homology to CHO from *Aspergillus* spp. (EU302818.1, [92, 93]), that possess endo-chitinolytic properties and CHO3 is phylogenetically related to the chitosanase found in *F*. *oxysporum* (EGU78186.1, [94]), that is involved in virulence (S6A Fig). Expression analysis of all identified chitosanases during mycoparasitism revealed their importance especially during late stages, when *T*. *atroviride* invades the host cells. Particularly when confronted with *R*. *solani*, transcription of *cho1- cho3* and *cho5* was significantly upregulated (~ 60-fold) and might therefore be important for mobilization of chitosan from the host cell wall (Fig 6B and S6B Fig). A significant but lower induction of expression of these enzymes was also observed for the ‘after contact’ condition with *S*. *sclerotiorum* and *B*. *cinerea*, two ascomycetic hosts. Interestingly, non-antagonistic growth conditions by confrontation of *T*. *atroviride* with itself (control condition) also resulted in moderate transcriptional upregulation of *cho1* to *cho3* (Fig 6B), which is a strong indication for the presence of chitosan also in the CW of *T*. *atroviride*.

### Cell wall chitin and chitosan affect the mycoparasitic attack and resistance to hosts

**Fig 7:**
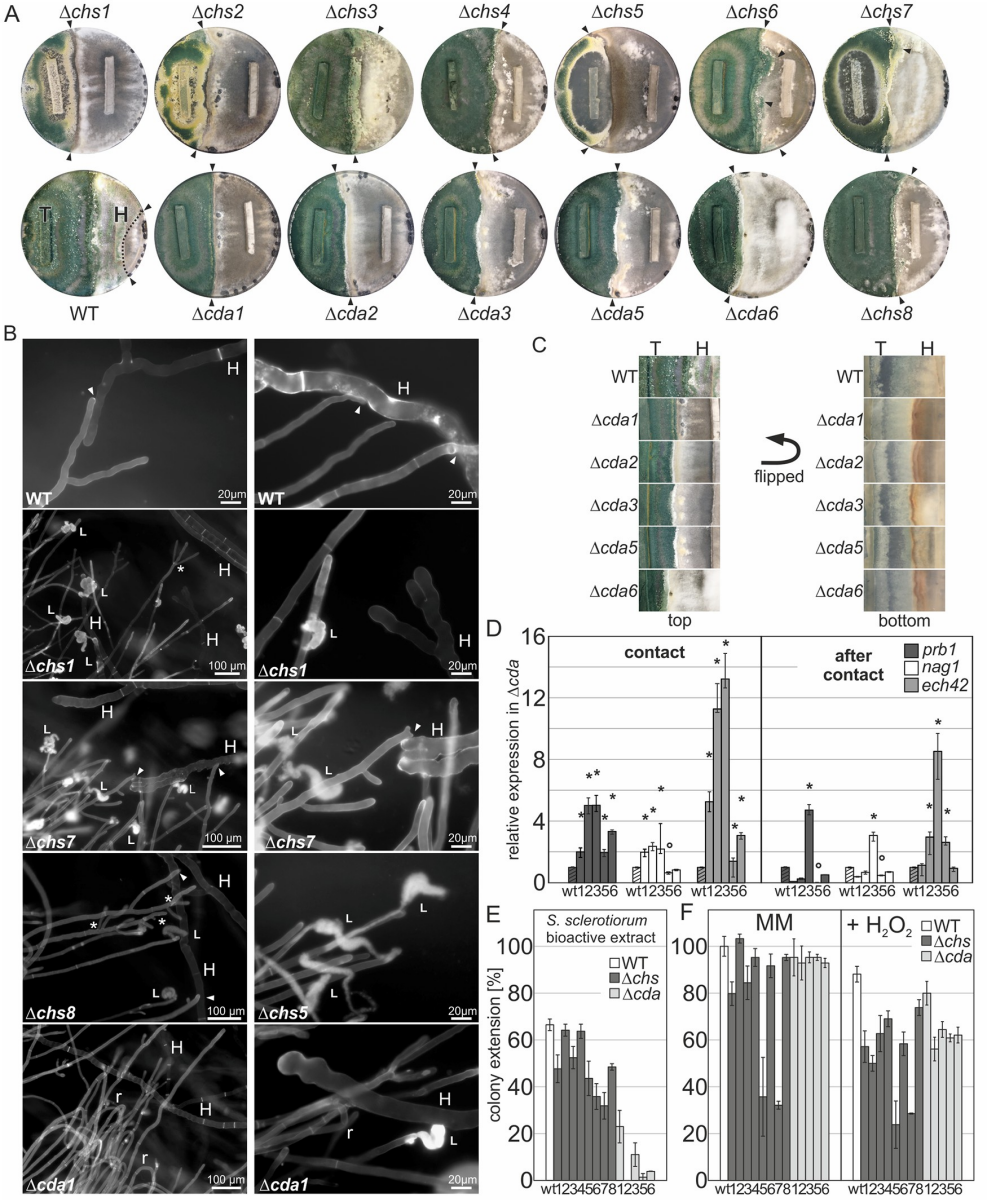
Cell wall chitin and chitosan affect the mycoparasitic attack and resistance to hosts. (A) Dual confrontation assays: A 5 x 30 mm slice of fully overgrown PDA plates of the indicated *T*. *atroviride* WT and mutant strains (T) was placed on the left side of a PDA plate 5 cm apart from a slice of the host *S*. *sclerotiorum* (H, right side) and incubated for 5 days. Arrows indicate the zonal overgrowth (dotted line) by *T*. *atroviride* over *S*. *sclerotiorum*. (B) Microscopic analysis of the confrontation zone of. *T*. *atroviride* WT and selected mutant strains against *S*. *sclerotiorum* using CFW staining in the inverted agar method for live-cell imaging [91]; arrows indicate hyphal attachment; L, hyphal leakage; r, hyphal retreat; *, increased hyphal branching; scale bar indicated. (C) Plates from dual confrontation assay in (A) were photographed from the bottom to show the brownish contact zone, in *cda* mutants compared to the wild type. (D) Relative expression of mycoparasitic indicator genes, *prb1*, *nag1*, *ech42* in the WT (wt, striped columns) and *cda* deletion (12356) mutants against *S*. *sclerotiorum*. Samples were collected at contact and after contact (4 mm overgrowth). WT alone (control condition) was arbitrarily set to ‘1’, (wt, striped columns). Expression data was normalized to the housekeeping gene *sar1*. Data were generated from two independent experiments and three technical replicates. Mean +/- SEM and p>0.005 are indicated with *, upregulated; °, downregulated. (E) Effects of antifungal metabolites and secreted enzymes from the host *S*. *sclerotiorum* on growth of *T*. *atroviride* WT and mutant strains. *S*. *sclerotiorum* was cultured on top of a diffusible cellophane membrane covering PDA plates and after 48 h of growth the cellophane disc was removed and plates containing the secreted active compounds were inoculated with an agar plug of *Trichoderma* WT and mutant strains. The percent growth inhibition by *S*. *sclerotiorum* secreted active extract was calculated from comparison with the growth of the respective strains on fresh PDA. A representative of three independent experiments is shown. (F) The susceptibility to oxidative stress of *Δchs* and *Δcda* strains was demonstrated on a solid medium containing H_2_O_2_. Colony extension in percent of the parental strain on minimal medium with (right panel) or without (left panel) the addition of 0.0075% H_2_O_2_ and growth for 72 h was determined. WT (white), chitin synthase mutants (grey), chitin deacetylase mutants (light grey) were compared. Mean +/- SEM from two independent experiments is shown.

In order to assess the mycoparasitic activity of mutant strains we monitored their capacity to overgrow *S*. *sclerotiorum*, *B*. *cinerea* and *R*. *solani*. We therefore investigated the mycoparasitic interaction in dual-plate confrontation assays, in which *T*. *atroviride* strains (antagonists) were inoculated at a defined distance from one of the three hosts. After recognition, *Trichoderma* spp. hyphae attach to and coil around the host hyphae and hook-like appressoria penetrate the cell wall, which is followed by growth of the antagonist inside the host [95]. All *cda* knockout lines demonstrated severe defects in overgrowth of *S*. *sclerotiorum* (Fig 7A) and *B*. *cinerea* and less pronounced with *R*. *solani* (S7A Fig). This behavior of *T*. *atroviride* to parasitize closely related phytopathogenic Ascomycota [96, 97] is considered to be the major trait that sets *Trichoderma* apart from the other mycoparasitic *Hypocreaceae* that mainly attack *Basidiomycota*. Interestingly, while the parental strain was able to entirely overgrow *S*. *sclerotiorum* within 5 days (overgrowth is marked with a dotted line), the *Δcda* mutants completely stalled growth already during the early phase of interaction, i.e. growth towards the host fungus and establishment of direct contact (Fig 7A). A distinct contact zone was particularly seen in *Δcda1* confrontations, which was also supported by microscopic analysis (Fig 7B). However, most *cda* deletion strains were able to overgrow *S*. *sclerotiorum* after prolonged incubation (14 days), suggesting that the deletion of *cdas* results in reduced chasing, coiling, and perhaps inability to invade the host. A strong defect in mycoparasitism was also observed for most of the *chs* mutants, with Δ*chs5* and Δ*chs1* being the most severely compromised KO strains. Again, mutants are delayed at contact and penetration of the hosts. A submerged growth towards the host was also observed indicating a loss of osmotic stability in such mutants. Thus, at contact, the tested Δ*chs1/2/5*/*7* are almost avirulent against *S*. *sclerotiorum* (Fig 7A).

To obtain a more detailed picture of the contact zone, microscopic analysis was performed on selected *chs* and *cda* deletion mutants that macroscopically showed the most prominent phenotype in the confrontation assay (Fig 7B). While the wild type attached and lysed the host *S*. *sclerotiorum* (H) the investigated mutants were unable to fully attack the host. Δ*chs5* and Δ*chs7* did not invade the host upon contact. Furthermore, Δ*chs1* and the myosin motor domain mutants Δ*chs5* and Δ*chs7*, but also Δ*cda1* showed massive hyphal leakage (examples of deflated cell contents are indicated, Fig 7B(L)). The Δ*chs1* strain responded to host stress with extensive clamydospore development and in both, Δ*chs1* and Δ*cda1*, intra-hyphal growth caused by the fungicidal effect on hyphae was observed (S7B Fig). Δ*cda1* was blocked in growth or retracted (Fig 7B(r)) upon contact with the host, and often balloon-like structures were visible. In Δ*chs8* an exorbitant and irregular apical branching was observed (Fig 7B(*)). No direct attachment could be observed, but lysis of the host.

*Trichoderma* inhibits or kills a host by parasitizing on its hyphae through cell wall degrading enzymes like chitinases (e.g. *ech42*), proteases (e.g. *prb1*) and glucanases [95]. Interestingly, although *cda* mutant strains seemed to be strongly compromised in host invasion, it seemed that they increased their production of lytic enzymes as a compensatory mechanism, which was visible by a browning of the growth medium most probably from generated oxidation products (Fig 7C). Transcriptional profiling of the mycoparasitic indicator genes supports this hypothesis by showing significantly higher *prb1* and *ech4*2 transcript levels in *Δcda1-6* than in the WT (Fig 7D). Expression of *ech42* was strongly induced at contact with *S*. *sclerotiorum* (∼13-fold) as well as *prb*1 expression in the *Δcda2* and *Δcda3* mutants (~5-fold). Expression of *nag1* (N-acetyl-glucosaminidase), and after contact also *prb1*, were strongly downregulated in *Δcda5* clones, indicative for a synergistic regulation of *cda*5 and these enzymes. Importantly, the absence of *cda3*, that was found massively induced during overgrowth of *S*. *sclerotiorum* (Fig 6A), resulted in a sustained induction of all indicator genes (Fig 7D), confirming its critical role in mycoparasitism.

Next we wanted to distinguish between an increased production of lytic enzymes by the chitin deacetylase mutants and their susceptibility to metabolites or active compounds, which are produced by the host and secreted into the growth medium. First all mutant strains and the wild type were grown on PDA plates covered with a cellophane disc to investigate the effect of secreted enzymes (and smaller diffusible factors) on the growth of the host (details are described in Materials and methods). *S*. *sclerotiorum* was placed on the plates after removal of the *T*. *atroviride* strains and growth was monitored.

As expected, *S*. *sclerotiorum* was highly susceptible to diffusible factors produced by the WT. Interestingly, growth was completely abolished on plates from mutant strain diffusible active compounds with the exception of Δ*cda6* (58.5% +/-6.9), Δ*cda5* (44.7% +/-5.9%) and Δ*chs8* (26.7% +/- 5.1), were growth of *S*. *sclerotiorum* was better compared to growth on the WT exudates (4.2% +/-1.9, and S7C Fig). Δ*chs3* (2.3% +/-0.4) had comparable growth to the WT. These results corroborate our findings and indicate that a perturbance of the cell wall composition results in elevated secretion of diffusible factors as defense strategy. Since cellophane allows passage of enzymes and small molecules an increase of secondary metabolite production is also very likely. In a second test series the susceptibility of mutant strains towards *S*. *sclerotiorum* secreted secondary metabolites and enzymes was tested. WT growth was reduced to 66% by the presence of exudates of *S*. *sclerotiorum* compared to growth on fresh PDA and growth of Δ*chs* strains was further decreased to 30–45% (Fig 7E). Strikingly, the Δ*cda* strains showed a pronounced growth reduction by 80 to nearly 100 percent. Thus, the deletion of the chitin synthases and deacetylases might affect cell wall composition in general leading to increased porosity with a negative impact on their resistance to extracellular active compounds produced by the host.

We further tested the sensitivity of the mutant strains towards H_2_O_2_ to simulate the presence of ROS (Fig 7F). In the past years the generation of reactive oxygen species in plant-pathogens, such as *B*. *cinerea*, *M*. *oryzae*, *Claviceps purpurae and S*. *sclerotiorum*, was discovered as conserved mechanism in virulence, infectious structure- and sclerotia formation and it has been speculated that ROS also serves as defense strategy during a mycoparasitic attack, similar to plant defense systems [78, 98–101]. Interestingly, growth of the chitin deacetylase knockout strains was most severely affected on plates containing H_2_O_2_ but also all Δ*chs* mutants showed decreased growth rates in comparison to the control condition (Fig 7F). Since most of the *cda* genes and *chs2*, *chs5*—*chs8* were also highly induced during ROS treatment in the wild type (Fig 3A) we conclude that chitin and especially chitosan play a critical role in scavenging ROS, which is putatively produced by the host as defense mechanism, and might contribute to the reduced virulence observed in the confrontation assays.

## Discussion

Data on how metabolic systems work together to build and modulate cell wall components to equip *Trichoderma* with its mycoparasitic activity is scarce. In particular, characterization of chitin synthesis and its regulatory systems remain largely enigmatic. Given the importance of chitin for fungal cell wall integrity and survival and the abundance of *chs* genes in the *Trichoderma* genome (Fig 1), our in-depth characterization of fungal chitin and chitosan formation sheds light on a biotechnologically highly relevant aspect of fungal activity.

We provide evidence that all eight chitin synthases of *T*. *atroviride* are part of a complex, intricately regulated chitin manufacturing machinery. The most important chitin synthases for vegetative and asexual development are the division one (*chs*1-3) and MMD containing synthases *chs5* and *chs7*. These class V and VII chitin synthases are highly conserved (S1 Fig) and critical for the co-delivery of not only chitin- but also glucan synthases to the plasma membrane along actin filaments [28, 79]. They promote specialized cellular processes associated with polarized growth and branching. MMD-CHS are only found in filamentous fungi and gene deletions showed strong and nearly lethal phenotypes in phytopathogenic fungi [38, 79], which we confirmed in *T*. *atroviride* (Fig 4A). Both MMD-CHS mutants further had strongly reduced chitin levels, a severe polar growth defect and an aberrantly high number of septa, which might be caused by a failure in transport of the remaining chitin synthases (Fig 5 and Table 1). Deletion of division 1 chitin synthases *chs1* to *chs3* seems to critically contribute to vegetative development but only *chs2* upregulation seems to compensate also for cell wall stress (Fig 3A). Moreover, environmental stimuli trigger remodeling processes in the cell wall, including chitin and chitosan rearrangements to protect the fungus in hostile environments [72, 102]. For example, we identified the class VI CHS6 as an exclusive osmo-responder (Fig 3A). Interestingly, this gene is located directly next to a HSP88 homolog and a fungal specific transcription factor that might regulate its expression upon osmotic changes (Fig 2B). In other fungi the roles of class VI synthases are very diverse and not conserved among different species [52]. Different from *C*. *albicans* and *A*. *fumigatus* [76] Ta*chs* are not transcriptionally activated upon treatment with the echinocandin Caspofungin, but interestingly *cda* transcription was affected (Fig 3A), indicating that the cell wall rigidity is changed due to deacetylation which might counteract the loss or decrease of β-1,3-glucan [72]. *In silico* analysis (Fig 3B) of the eight chitin synthase promoters with known response elements that confer binding of stress and development related transcription factors in *S*. *cerevisiae* provided further evidence that external stimuli have a high impact on transcriptional regulation of the chitin processing enzymes. Strong response to ROS and mycoparasitism related stresses coincided with a higher enrichment of such elements. Interestingly, among the binding motives that were found highly enriched was also the Msn2/Msn4 homologous AGGGG-binding element that is recognized in *T*. *atroviride* and *N*. *crassa* by the transcription factor Seb1 [81, 103] and is related to HOG-stress and nutrient availability. Moreover, a highly conserved enrichment of the TEC1 binding motif in the promoters of *chs5* and *chs7* was identified. In *T*. *atroviride* Ste12/Tmk1 govern processes in vegetative development and mycoparasitism [104]. For Tec1, which is the co-factor of the Ste12 in yeast no orthologue has yet been identified in *T*. *atroviride*. Using the sequence of the Tec1 homolog in *A*. *nidulans*, AbaA, we were able to identify a distantly related homolog, pID322845. If this protein is indeed involved in stress related transcriptional regulation of chitin synthesis remains to be determined.

Chitin synthase activity can also strongly depend on posttranslational activators, such as ScChs4/ScSkt5, which serves as a ScChs3 (class IV) enhancer [105, 106]. Moreover, correct transport through chaperons (e.g. ScChs7/ NcCse7 [83, 87]), association with adaptor proteins (e.g. ScCHS5 [82, 84]), as well as phosphorylation and/or prenylation [107] are important players in regulation. Among these (additional) regulators, the ScChs7 chaperone function seems to be crucial to mediate ScChs3 activity, which has been confirmed also in *N*. *crassa* and *F*. *oxysporum* [87, 88]. Interestingly, in contrast to TaΔ*chs4*, which revealed a less pronounced phenotype, the absence of the Sc*CHS5* and Sc*CHS7*/Nc*cse*7 orthologs, Ta*cse5* and Ta*cse7*, led to an increased branching phenotype, probably due to altered chitin deposition (Fig 5A). In addition, the formation of an aerial mycelium indicates an osmotic imbalance due to the observed altered cell wall chitin content, and evidenced in particular by the strong inhibition with CFW. The fact that the deletion of these genes leads to a stronger growth defect than observed for their corresponding chitin synthase (*chs4*), suggests an involvement of these chaperons in the secretion of other chitin synthases [88]. For now, we cannot rule out that it is a secondary effect concerning exocytosis. Future studies are needed to enhance our understanding on the interaction of these auxiliary proteins with other chitin synthases.

Transglycosylases (CRHs) play an essential role in branching and cross-linking of glucan with chitin, which adds to the structural integrity of the cell wall. We identified two CRH1 and one CRH2 orthologues in *T*. *atroviride*, the latter is considered to be the leading contributor to chitin-glucan synthesis in *S*. *cerevisiae*. In *T*. *atroviride* transcription of all *crh*s was significantly decreased during vegetative hyphal network formation (48 h) upon *chs* deletion, especially in the ΔMMD-*chs* strains. Thus, the proposed strong decrease in cellular chitin seems to negatively affect also transcription of the crosslinking enzymes (Fig 4E). Interestingly, the transcriptional levels of *crh1a* and *crh2* considerably increased in the Δ*cda* strains (Fig 4F). It was shown that the nascent chitin chains are transferred directly after extrusion to the periplasm to the glucan matrix as acceptor, which in turn increases solubility of the chitin-glucan complexes [89, 90]. The higher expression of the *crhs* in the chitin deacetylase mutants might therefore hint at a compensatory action of these crosslinking enzymes to increase solubility of the otherwise too crystalline chitin.

Mycoparasitism includes shaping infection structures as well as the production of antimicrobial secondary metabolites [108, 109], which facilitate penetration of plant pathogens. Our analysis of cell wall remodeling enzymes in *T*. *atroviride* during mycoparasitism on three different hosts revealed that a concerted action of chitin synthesis and deacetylation that differs greatly from the vegetative transcriptional profile is involved in the parasitic attack. Intriguingly, all of the created knockout mutants were severely compromised in mycoparasitism, with the ΔMMD*-chs*, Δ*chs1* and Δ*cda1* most severely affected. Two high responders, *chs8* and *cda1*, were identified, while transcription of *chs1*, 4, 5 and 7 was only moderately induced and *chs2* and *chs6* levels did not change (Fig 6A). Interestingly, *chs8*, which shows also homology to hyaluronan synthases, is located downstream of *cda1* and a gene encoding an UDP-N-acetylglucosamine 6-dehydrogenase (UNGD, manuscript in preparation). This gene cluster is conserved among fungi harboring this special type of chitin synthase [53, 57, 58]. In contrast to other chitin synthases, such chitin/hyaluronan synthases may sequentially use two different sugar monomer substrates (UDP-N-acetyl-D-glucosamine and UDP-D-glucuronate [110]) to produce either hyaluronan or chitin [111]. We therefore speculate that during interaction with the host a complex cell wall remodeling takes place and CHS8, together with CDA1, forms a protective chitin glycopolymer layer, which might lead to increased resistance. For instance, a cell surface protective function of human-epithelial cells against *Candida* infections is mediated by extracellular hyaluronan [112]. Although CHS8 homologs are present in some fungi with parasitic life styles, the enzyme has only been characterized in the phytopathogen and closely related *F*. *graminearium*. The disruption of Fg*chs8* also led to strongly impaired virulence [56].

*Trichoderma* inhibits or kills the host by breaking down its hyphae through cell wall degrading enzymes like chitinases, proteases and glucanases [95]. *T*. *virens* and *T*. *atroviride* contain the highest number of genes encoding chitinolytic enzymes among described fungal genomes [9, 113, 114] and we showed only recently that transcription of *cho5* is regulated in a *tmk1* MAPK kinase dependent manner [115]. Here we demonstrate that all of the six identified chitosanases are highly upregulated during contact and feeding (after contact) of *T*. *atroviride* on the hosts (Fig 6B). It has already been shown that *R*. *solani* contains high levels of chitin in its cell wall [116] and regarding our new findings we speculate that a considerable amount of that might be present as chitosan. Interestingly, chitosanases were also highly expressed during *Trichoderma* self-contact, suggesting a more fundamental role in the cell wall remodeling process that might stretch beyond mycoparasitism. For chitinases, such an additional function, apart from mycoparasitism, has already been described [21]. We plan to decipher the distinct roles of chitosanases in the self and non-self recognition process, and hope that such insights will further enhance our understanding of biocontrol mechanisms.

It is likely, that successful mycoparasites such as *Trichoderma* suppress the host recognition and defense system via use of their chitin/chitosan remodeling enzymes; one of the most relevant activities of CDAs was reported in plant–pathogen interactions during penetration [50]. In several fungal species chitin deacetylation is essential for cell wall rigidity and for resistance to chitinolytic enzymes. Cell wall chitosan in phytoparasites contributes to their resistance against hostile (endo-) chitinases, an involvement of CDAs in mycoparasitism was therefore strongly suggested. Phylogenetic analysis of published CDAs from other fungi with well-characterized functions showed that the CDAs can be grouped into four different clades (Fig 1C). Interestingly, a very large group of fungi contains CDAs (*P*. *chlamydosporia C*. *lindemuthianum*, *P*. *graminis*, *Pestalotiopsis sp*. and *M*. *oryzae*), that all play a major role in virulence [43, 61, 62]. They are extracellular, contain a secretion signal peptide, and some of them have already been shown to be involved in different parasitism strategies [117]. TaCDA1 and TaCDA5 group to this clade most closely related to *M*. *oryzae* MggCBP1 and MggCDA2 and *P*. *chlamydosporia*, hinting at their importance in virulence. In fact, Δ*cda1* and to a lesser extent Δ*cda5* strains displayed severely compromised mycoparasitic capabilities. In the wildtype strain, expression levels of *cda1* and *cda5* were highly elevated during mycoparasitism and oxidative stress caused by ROS. Moreover, the CBMs present in these enzymes are important for substrate recognition of oligosaccharides and are implicated in enhancing deacetylase activity by increasing the accessibility of the substrate to the catalytic domain [62, 118]. TaCDA3, TaCDA4 and TaCDA6 do not group with any of the other deacetylases, except for their *T*. *reesei* CDA4 orthologue. Given their expression during vegetative development and self-recognition in the confrontation assays, they might be involved in distinguishing the own cell wall from that of the host fungus. CDA4 and CDA6 are highly homologous, which is a strong evidence for gene duplication that increases genetic variability and may contribute to adaptability in a changing environment [119]. However, we were not able to delete *cda4*, pointing at a different but yet crucial role of this gene in vegetative development. Regarding mutant morphology, the conidia of the Δ*cda6* mutant seemed more crystalline in comparison to the WT or other mutant strains. Intriguingly, *cda3* was only expressed during spore formation, and the Δ*cda3* mutant produced also considerably less conidia while hyphal development was only mildly compromised. Since the predicted protein does neither have a signal peptide, nor a fully functional deacetylase domain it is plausible that this protein evolved as a regulatory protein rather than an active enzyme. Taken together, our data suggests a critical involvement of these enzymes in (conidial) CW maturation.

The third group of CDAs in the phylogenetic analysis harbors a transmembrane domain and is needed for vegetative development. *Amylomyces rouxii*, *C*. *neoformans* and the two CDAs of *S*. *cerevisiae* critical for spore formation are present in this group [41, 63–66]. No CDAs from *Trichoderma* were found directly connected with this branch, but in the adjacent branch all proteins harbor a transmembrane helix at their N-terminus, including *Trichoderma* CDA2 (Fig 1C). Preliminary experiments also detected TaCDA2 bound only to the cell wall or membrane but not secreted into the culture filtrate (Gruber and Kappel, unpublished results). Given the constitutive expression of *cda2* together with *chs3* throughout the whole cell cycle, these enzymes might act in a tandem mechanism to maintain a certain level of deacetylation of the nascent chitin chains or even chitosan, which has been proposed earlier [41]. Δ*cda2* deletion mutants showed a dysregulated apical dominance (Fig 5A), which has been observed in a large number of fungi [120, 121] presumably in response to the abnormal accumulation of exocytic vesicles at the hyphal tip. We believe that elucidating the specific role of the six CDAs will greatly enhance our understanding of cell wall formation in *T*. *atroviride*.

Even though most of the *cda* mutants have a strong mycoparasitic defect, they could at least partly overgrow the host (Fig 7A). We hypothesize that this is due to a reduced protection of the hyphal cell wall during this interaction. To this end, we observed a robust transcriptional induction of lytic enzyme production as compensatory means to evade the counterstrike by the host in the chitin deacetylase mutants (Fig 7D). However, this still failed to fully restore the mycoparasitic capability. Remarkably, some of these enzymes are up-regulated even before contact and during overgrowth of the host fungus in the wild type [114, 122]. With ROS being another potent effector in phyto- and mycoparasitism [78, 101] and chitosan being implicated in ROS scavenging [123] we looked at the influence of ROS upon growth of these strains. Consequently, ROS hampered growth of all deletion mutants, especially the Δ*cda* strains (Fig 7F). Production of ROS throughout different growth stages of *S*. *sclerotiorum* was demonstrated [101] and it is noteworthy that susceptibility of the *Trichoderma* mutants to metabolites or enzymes produced by the host *S*. *sclerotiorum* was increased in the Δ*cda* strains, but less pronounced in the Δ*chs*. All of these findings therefore provide the first evidence that chitosan is important in the mycoparasitic attack by *T*. *atroviride* and apart from serving as simple disguise towards hostile chitinases and chitin receptors might decrease the reactive oxygen burden and/or protect against other diffusible active compounds. Since the Δ*cda* strains contain similar amounts of chitin as the WT this might be due to differences in the chitosan levels of the mutants. It will be necessary to determine the exact amounts of chitosan in *T*. *atroviride* and mutant strains and exact nature of diffusible factors secreted by the plant-pathogens during mycoparasitism to corroborate our hypothesis.

**Fig 8:**
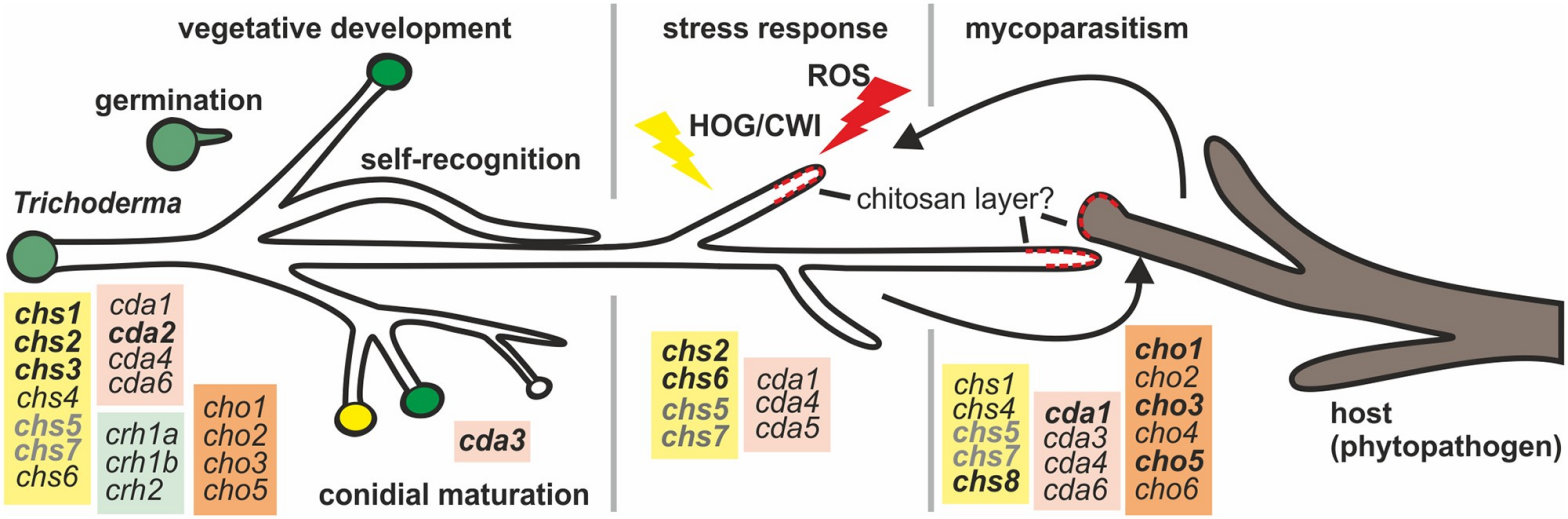
Specified roles of chitin-modifying enzymes during the life cycle of *T*. *atroviride*. Schematic representation of the involvement of chitin synthases (*chs*, yellow), chitin deacetylases (*cda*, pink), chitosanases (*cho*, orange) and transglycosylases (*crh*, turquoise) in various stages of development of *Trichoderma atroviride*. Vegetative development, including germination, conidiophore formation, conidial maturation and self recognition, environmental stress response and mycoparasitism with invasion and feeding on a host cell wall, are depicted. The most important players are depicted in bold letters. The role of putatively secreted and membrane-bound enzymes in mycoparasitism (in association with secondary metabolites and other enzymes, not shown), exogenous chitin and chitosan degradation (released by the fungi), and in the remodeling and recycling of the own cell wall (self-recognition) and the host is demonstrated.

*Trichoderma* derived products are already used in agriculture but there is still a considerable need to enhance their applicability in crop control, particularly towards plant pathogens. This demands the extension of our knowledge on how *Trichoderma* manages to bypass the host defense mechanisms, for cell wall penetration and ultimately host degradation. Our results elucidate critical aspects of cell wall synthesis and remodeling in mycoparasites (Fig 8). Five out of eight chitin synthases (*chs*1, 2, 3, 5 and 7) are non-redundant and indispensable for mycoparasitism and play critical roles in chitin biosynthesis during vegetative development. Our findings further highlight the importance of chitin deacetylases for the plasticity of the cell wall throughout the whole vegetative development and in asexual maturation. Even more important for aspects of biocontrol CDAs are essential for mycoparasitism. The excessive production of six newly identified chitosanases during mycoparasitism but also self/non-self-recognition processes is especially intriguing and expands the enzymatic repertoire known from *Trichoderma* spp. This implies that the first line of attack engaged by *Trichoderma* involves chitin *and* chitosan degrading enzymes that compromise the host cell wall integrity by targeting also its chitinous backbone. Hence, cell wall construction of the hosts will be another issue that has to be considered in future studies.

## Materials and methods

### Strains and cultivation conditions

*T*. *atroviride* IMI206040 (teleomorph: *Hypocrea atroviridis*, (http://genome.jgi-psf.org/Triat2/Triat2.home.html) maintained on potato dextrose agar (PDA, BD, Franklin Lakes, USA), incubated at 28 °C with a 12h/12h light/dark cycle. Chemicals were obtained from Roth (Karlsruhe, Germany), Sigma (Sigma Aldrich, St. Louis, MO) and enzymes and kits were obtained from Thermo Fisher (Thermo Fisher Scientific, Waltham, MA USA) and Bio-Rad (Bio-Rad Laboratories, Hercules, CA, USA). For assessing the growth rate of WT and mutant strains mycelial agar plugs were placed on PDA, with or without hygromycin B (200 μg/ml) and incubated for 72 h. In order to test susceptibility towards CW disturbing agents and HOG on solid medium, PDA was supplemented with 200 μg/ml Calcofluor white (Fluorescent brightener 28, CFW), 150 μg/ml Congo red (CR) or 1.2M sorbitol. Sensitivity to oxidative stress provoked by hydrogen peroxide (0.0075%) was tested on SM medium [124] (2% agar agar, 2% glucose). Radial growth was measured and captured every 24 h. Conidial maturation, spore size and concentration were investigated after 7 days on PDA. Conidia were harvested in a defined volume of 0.9% NaCl /0.05% Tween-80 solution and counted with a Tecan reader (Spark multimode microplate reader, Tecan Group Ltd., Männedorf, Switzerland) in a cell counting chamber (Cell Chip, NanoEnTeK, Tecan). Conidia from different maturation stages were prepared as described in [70] for RNA preparation. For shake flask cultivations SM with 0.05% peptone and 1% glucose was inoculated with 1×10^6^ conidia/ml and cultivated at 28 °C and 250 rpm. To investigate the stress induced transcription replacement studies [70] were performed by transferring pre-cultures (WT) from liquid medium grown for 24 h to medium with different stressors: 10% and 40% glucose, 10% glycerol, 0.7 M NaCl, 1.2 M sorbitol, 0.025% SDS, 0.75% H_2_O_2_, 150 μg/ml CR, 200 μg/ml CFW, 15 μg/ml Caspofungin (CF). After 1 h and 24 h mycelia were harvested for RNA extraction and biomass determination, respectively.

### Generation of knockout strains—Genetic manipulation of *T*. *atroviride*

Knockout lines were established using the split marker technique [125] with an *E*. *coli hph*- phosphotransferase marker cassette. The *hph* gene marker cassette is under control of the *Trichoderma pki1* promoter and *cbh2* [126] terminator, derived from plasmid pan7-1 [127] and cloned in pBluescript II KS/SK- backbone (unpublished). Primers (F1, F2, F5 and F6; S4 Table), were designed to amplify around 1.5 kb fragments up-und downstream of the ORF of the target gene. Primer combinations HY/F4 and F3/YG amplify half of the 5´or the 3´ regions of the *hph* gene plus 1.5 kb of the target gene 5´or 3´flanking region, respectively [125]. Purified single PCR-fragments were subjected to double joint PCR reaction [128] with primer combination F1 and YG for the 5´ part, and HY and F6 for the 3´part, respectively. All PCR reactions were performed using the Phusion High-Fidelity DNA Polymerase (Thermo Fisher). Cassettes were fused in one single reaction and purified (to avoid unspecific integration), using the GeneJET Gel Extraction Kit (Thermo Fisher). Purified cassettes in equimolar amounts were used for protoplast [129] and spore mediated fungal transformation, which was adapted for *T*. *atroviride* from a protocol described for *T*. *reesei* in [130]. Briefly, freshly harvested spores were incubated in YPD (1% (w/ v) glucose) at 30°C, 300 rpm for 6 hours for conidial swelling, then carefully washed with decreasing amounts of 1.1 M ice cold sorbitol until a final volume of 75 μl per reaction. Split marker inserts (3–6 μg) were added and subjected to electroporation by use of the Biorad MicroPulser Electroporator (Bio-Rad) with 1.8 kV for ca. 5.5 ms. Regeneration followed with YPD/ 1.1 M sorbitol over night at 28°C before plating on PDA with 200 μg/ml hygromycin B. Positive transformants were confirmed by PCR. A set of PCR amplifications (KO_Fw/KO_Rv; combination with YG and HY, S4 Table) was used to discriminate homologous recombination from ectopically inserted constructs, parental genotypes or heterokaryons (S4D Fig). Candidates were purified employing single spore isolation until mitotic stability. As mock control, for all experiments the empty hph plasmid was transformed into the WT.

Complementation strains (*ReChs or ReCda)* were generated by reintroducing the WT sequence flanked by ca. 1,600 bp native promoter and terminator sequences into the genome of the mutant by random integration or gene replacements by co-transformation with plasmid p3SR2 [131]. Transformants were selected on acetamide containing medium and purified by single spore isolation (S5A and S5C Fig). A list of all mutant lines generated in this study is presented in S6 Table.

### Isolation and purification of DNA

A rapid DNA purification protocol [132] and an isolation protocol for highly purified genomic DNA were used [133].

### Dual confrontation assays

Confrontation assays of *T*. *atroviride* WT, *chs* and *cda* deletion strains against *Rhizoctonia solani*, *Botrytis cinerea*, and *Sclerotina sclerotiorium* were performed on PDA as described in [104] with approximately 5 cm distance of parasite and host. Plates were incubated for five days in circadian illumination and photographed. As controls, *T*. *atroviride* confrontation with itself or non-challenged *T*. *atroviride* were used. For RNA extraction confrontation assays were performed on PDA covered with sterile cellophane discs. *T*. *atroviride* mycelia were harvested before contact (BC), and after contact (AC) and immediately frozen in liquid nitrogen.

### Growth inhibition by metabolites and secreted enzymes

Susceptibility to secreted secondary metabolites and proteins of *T*. *atroviride* and mutant strains was tested in plate assays according to [134] with some modifications. *S*. *sclerotiorum* was inoculated on PDA plates covered with a cellophane disc for 72 h in the dark at 25°C, allowing the produced metabolites to diffuse through the membrane into the agar. Note, that cellophane not only allows the passage of low-molecular-weight metabolites, but also active enzymes [135]. The cellophane disc was removed when the mycelium covered about 3/4 of the plate and an agar plug of *T*. *atroviride* wild type or mutant strains was placed in the middle of the plate to assess the mutants putatively altered susceptibility. The growth diameter was recorded after 52 h and growth inhibition was calculated in % compared to growth of the corresponding WT or mutant strains on fresh PDA. The same procedure was followed to investigate the secretion of metabolites and enzymes of *T*. *atroviride* WT and mutant strains and their potential to inhibit *S*. *sclerotiorum* growth. The antifungal activity against *S*. *sclerotiorum* was evaluated by comparing growth to fresh PDA plates in %. Data was generated from two independent experiments in both set-ups.

### RNA isolation and cDNA synthesis

For RNA extraction ca. 100 mg of mycelium was ground to a fine powder with glass beads and a bead mill (2x 60s at 6 m/s; Bead Ruptor 24 Elite Bead Mill Homogenizer, Omni International, VWR). Total RNA from liquid shaking culture and from spores was isolated using the guanidinium thiocyanate method [136]. When RNA was extracted from mycelium grown on plates the GeneJET Plant RNA Purification Kit was used. Isolated RNAs were treated with DNAse I, and purified with the GeneJET RNA Cleanup and Concentration Micro Kit. cDNAs were generated with the Revert Aid H-minus cDNA synthesis kit, using 1 or 5 μg RNA. All kits and enzymes for RNA purification and cDNA synthesis were purchased from Thermo Fisher.

### Gene expression analysis

qRT-PCR reactions were performed in a Biorad iCycler iQ (Bio-Rad) and a Rotor-Gene 6000 (QUIAGEN, Venlo, Netherlands) as described previously [137]. Primers and protein IDs from the DOE Joint Genome Institute database are listed in S5 Table. Relative gene transcript levels were quantified and normalized to the corresponding signals of *sar1* (differentiation between high sequence homologies of *T*. *atroviride* and hosts in confrontation assays), and *tef1* [138, 139]. The fold change relative to the control conditions was calculated using the ΔΔC_T_ method [140] with REST software [141]. All samples were analyzed from at least two independent experiments with three technical replicates.

### Hyphal GlcNAc content assay

To measure the chitin content in the cell wall of wild type and mutant strains the protocol described by [27] was adapted for *Trichoderma atroviride*. Strains were grown on PDA plates covered with a cellophane disc until a diameter of approximately 2–2.5 cm was reached. 40–50 mg mycelium was harvested into tared 2-ml microcentrifuge tubes containing 2 large and 100 μl small glass beads (2.85–3.45 and 0.75–1.0 diameter, respectively) and dried overnight to determine dry weight (typically 5–7 mg). The cell walls were extracted with 1 ml 6% KOH at 80°C for 90 min. Samples were centrifuged at 14,000 rpm for 20 min, and the supernatants were used to indirectly determine dry biomass via a protein content assay with Bradford reagent (Biorad). A linear correlation of protein content to biomass had been established with defined amounts of dry weight, previously. Pellets were washed 3x in 1 ml 1xPBS, and 1x with 1 ml of Mc Ilvaine’s buffer (0.2 M Na2HPO4, 0.1 M citric acid, pH 6.0) and frozen at 20°C or directly processed further. 5μl of Chitinase from *Trichoderma viride* lyophilized powder, ≥600 units/g solid enzyme mix (5 mg/ml in PBS, Sigma) were added to 200μl of Mc Ilvaine´s buffer to hydrolyze chitin to GlcNAc. Samples were incubated for 20–24 h at 37°C. For colorimetric determination the protocol given by [27] was followed.

### Microscopic analysis

Strains grown on PDA for 18 h were applied in a droplet of 30μl CFW stain (20μM, Sigma #F3543) with the inverted agar method [91] and imaged with an inverted Zeiss Axio Observer Z1 (Zeiss, Oberkochen, Germany) with differential interference contrast optics and 405nm excitation/ 430–470nm emission. Germlings were investigated on an Olympus CX33 (Olympus, Hamburg, Germany) after 0, 4, 8, and 16 h of germination in liquid PDB at 28°C, 300 rpm. Visualization and semi-quantification of *Trichoderma* cell wall chitin was performed using the following cell wall stains: Calcofluor White (CFW) M2R at a final concentration of 2 μM to non-specifically label β-1,4-glucans including chitin and Congo Red (CR, Sigma #C6767) at a final concentration of 50 μM to very specifically label α- and β-chitin [115]. Fluorescence signals were assessed using CFW and CR. All samples were incubated for 5 min before imaging. In order to compare hyphae from parental and mutant strains, all samples were measured by densitometry using the MacBiophotonics ImageJ work package available at (https://www.macbiophotonics.ca/software.htm) as described in [115]. Defined rectangular areas were measured in the Spitzenkörper and in the lateral cell walls of the subapical zone. On average 25–60 analysis were performed per hyphae.

### Transcriptional response element detection

Literature search for stress response elements for different cellular processes in fungi found four main groups whith specific elements: CWI (CDRE, RML1, crzA, Crz1p, CRZ1-sn2/4, CRZ1-2, CreA), DEV (ARE, AREanalog, Are2/Nit2, BrlA), HOG (Sko1p, AtfA-1) and ROS (Hap1, Yap1, AtfA-2,CRE-CRZ-Mn). Additional fungi jaspar matrixes, most due to yeast studies were used. Scanning of the *chs*1-8 promoters showed specific elements for groups of general stress (TEC1, HSF1, STP4) and oxidative stress (ROX1, SKN7, STB5, AFT2). For more details see S3 Table. For scanning of sequences against patterns the Regulatory Sequence Analysis Tools (RSAT) http://rsat-tagc.univ-mrs.fr/rsat/ was used. For the eight *chs* promoters (1,200 bp upstream) sequences against our own and jaspar patterns only hits with a score < 5 were discarded. Finally, overlapping motives and hits on the reverse strand were joined. Similar enriched motives in groups of *chs* promoters (e.g. *chs*5,7) were detected using RSAT. Additionally, the 50 most enriched motifs for the four condition groups CWI, ROS, HOG, FUS (here using *T*. *atroviride* and related species orthologues) were detected with Meme http://meme-suite.org/. The eight *chs* promoters from *T*. *atroviride* were then scanned with the 200 motives using RSAT. Further, a correlation to known jaspar motives was calculated for functional hints by RSAT.

### Bioinformatics and statistics

For transcriptional analyses, the hierarchical clustering algorithm Hierarchical Clustering Explorer 3 (HCE3) [142] with average linkage and Euclidean distance measure was applied and values were quantitatively illustrated using the grey scale scheme in Fig 2 and color scheme in Figs 3 and 6. The colors were selected based on the jet color mapping for the results of the function y = a*1/e^b^*^x^+c, where a, b, c are constants that were chosen so that value differences are easy to compare visually and x represents the relative expression value that was calculated with the REST software [141]. The values of a, b, c and the according formulas are freely available at https://github.com/mamut-m/expression-colormap. Statistical analysis for qRT-PCR was performed with REST software with a pair wise fixed reallocation randomization test. For all other experiments were statistical analysis was needed Student´s t-test was used, assuming unequal variance of groups. At least two biological and three technical replicates were used for statistical analysis. Phylogenetic analyses were performed in Mega7 [143], using Neighbor Joining, a distance algorithmic method. Stability of clades was evaluated by 1,000 bootstrap rearrangements. Structure/function prediction was performed using InterProScan [144], signalP 5.0 [145], and transmembrane domain prediction tool TMHMM 2.0 [67].

## Supporting information

S1 Fig Phylogenetic analysis of all identified *T*. *atroviride* chitin synthases and chitin deacetylases. (PDF)

S2 Fig Relative expression levels of chitin synthase and chitin deacetylase genes. (PDF)

S3 Fig Biomass formation under stress conditions after 24h. (PDF)

S4 Fig Growth, conidiation and verification of deletion of *chs* and *cda* genes. (PDF)

S5 Fig Rescue strains. (PDF)

S6 Fig Phylogenetic analysis of all identified *T*. *atroviride* chitosanases and their basal transcription levels. (PDF)

S7 Fig *T*. *atroviride* deletion mutants show reduced mycoparasitism on *B*. *cinerea* and *R*. *solani* while secretion of active compounds is increased. (PDF)

S1 Table *CHS4*-*CSA1* gene cluster. (PDF)

S2 Table *CHS6*-*CHS5*-*CHS7* gene cluster. (PDF)

S3 Table Identified promoter response elements. (PDF)

S4 Table Cloning primers. (PDF)

S5 Table qPCR primers. (PDF)

S6 Table Generated knockout and rescue strains. (PDF)
