# Supplementary material for: Chitin and chitosan remodeling defines vegetative development and Trichoderma biocontrol
Source: PLoS Pathog. 2020 Feb 20;16(2):e1008320. doi: 10.1371/journal.ppat.1008320 (PMC7053769; doi:10.1371/journal.ppat.1008320)
Supplement: S1 Fig — (PDF) [file ppat.1008320.s001.pdf]

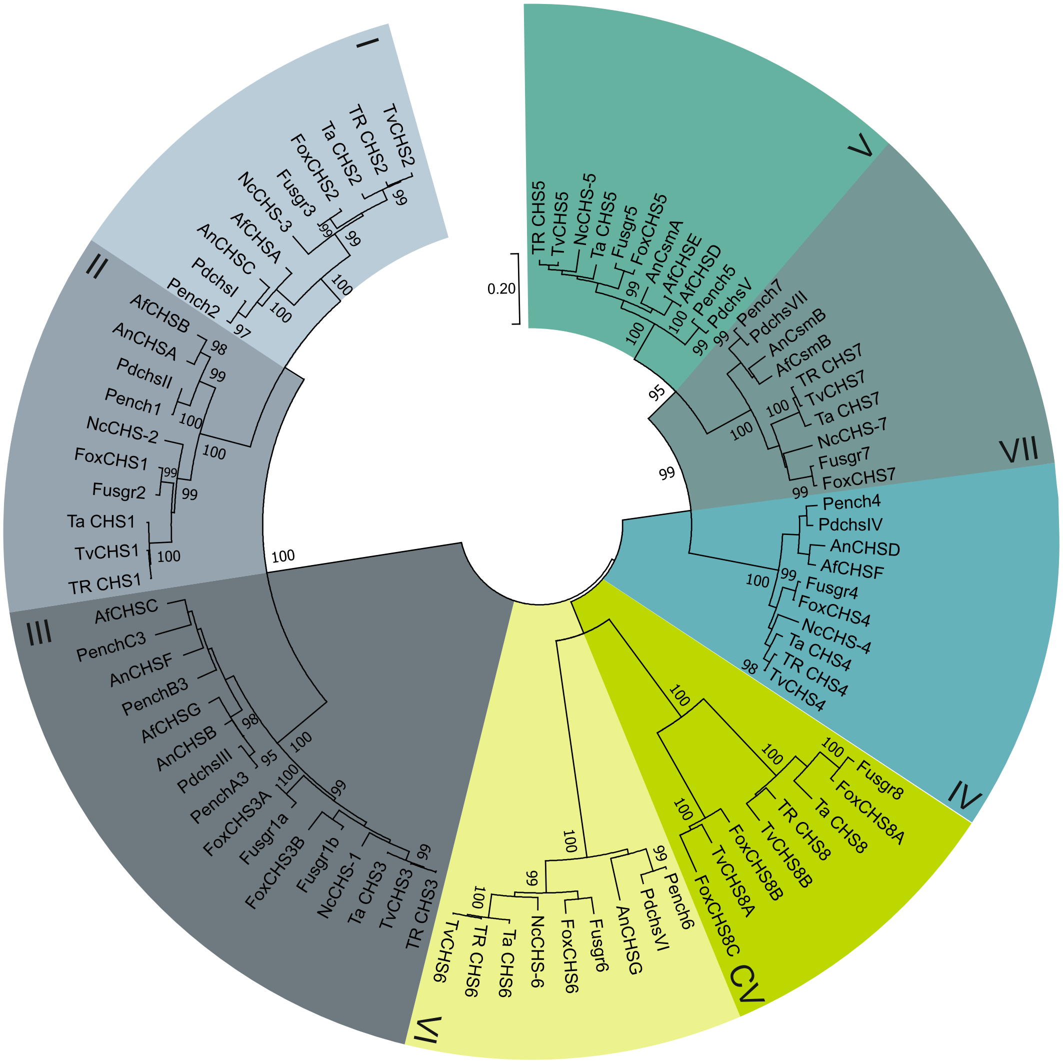
I
TvCHS2
TR CHS2
Ta CHS2
FoxCHS2
Fusgr3
NcCHS-3
AfCHSA
AnCHSC
PdchsI
Pench2
II
AfCHSB
AnCHSA
PdchsII
Pench1
NcCHS-2
FoxCHS1
Fusgr2
Ta CHS1
TvCHS1
TR CHS1
III
AfCHSC
PenchC3
AnCHSF
PenchB3
AfCHSG
AnCHSB
PdchsIII
PenchA3
FoxCHS3A
Fusgr1a
FoxCHS3B
Fusgr1b
NcCHS-1
Ta CHS3
TvCHS3
TR CHS3
VI
TvCHS6
TR CHS6
Ta CHS6
NcCHS-6
FoxCHS6
Fusgr6
AnCHSG
PdchsVI
Pench6
CV
FoxCHS8C
TvCHS8A
FoxCHS8B
TvCHS8B
TR CHS8
Ta CHS8
FoxCHS8A
Fusgr8
IV
TvCHS4
TR CHS4
Ta CHS4
NcCHS-4
FoxCHS4
Fusgr4
AfCHSF
AnCHSD
PdchsIV
Pench4
VII
FoxCHS7
Fusgr7
NcCHS-7
Ta CHS7
TvCHS7
TR CHS7
AfCsmB
AnCsmB
PdchsVII
Pench7
V
PdchsV
Pench5
AfCHSD
AfCHSE
AnCsmA
FoxCHS5
Fusgr5
Ta CHS5
NcCHS-5
TvCHS5
TR CHS5
0.20

S1 Figure. Phylogenetic analysis of all identified *T. atroviride* chitin synthases and chitin deacetylases.

Extended evolutionary tree representing the analysis on which the compressed tree shown in Figure 1A is based. The tree was generated by use of the whole sequence of the indicated enzymes with the phylogenetic analysis software MEGA7, 1,000 bootstraps and the neighbor-joining-method. Numbers next to nodes indicate the bootstrap value. The bar marker indicates the genetic distance, which is proportional to the number of amino acid substitutions. Fungal chitin synthase classes are indicated in roman numbers (I-VII and CV). The following complete or relevant enzyme sets were mined from respective databases for CHS and also for CDA analysis: Ar, *Amylomyces rouxii*; Af, *A. fumigatus*; An, *Aspergillus nidulans*; Cl, *Colletotrichum lindemuthianum*; Cn, *Cryptococcus neoformans*; Fusgr, *Fusarium graminearium*; Fox, *F. oxysporum*; Fv, *Flammulina velutipes*; Gb, *Gongronella butleri*; Mgg, *Magnaporthae oryzae*; Nc, *Neurospora crassa*; Pd, *Penicillium digitatum*, Pench, *P. chrysogenum;* Pa, *Podospora anserina*; Pb, *Phycomyces blakesleeanus;* Pc, *Pochonia chlamydosporia*; Pes, *Pestalotiopsis sp.*; Pgt, *Puccinia graminis*; Rs, *Rhizopus stolonifer (nigricans)*; Sc, *Saccharomyces cerevisiae*; Sch, *Schizophyllum commune*; Ta, *T. atroviride*; Tr, *T. reesei*; Tv, *T. virens*.
