# Supplementary material for: Chitin and chitosan remodeling defines vegetative development and Trichoderma biocontrol
Source: PLoS Pathog. 2020 Feb 20;16(2):e1008320. doi: 10.1371/journal.ppat.1008320 (PMC7053769; doi:10.1371/journal.ppat.1008320)
Supplement: S2 Fig — (PDF) [file ppat.1008320.s002.pdf]

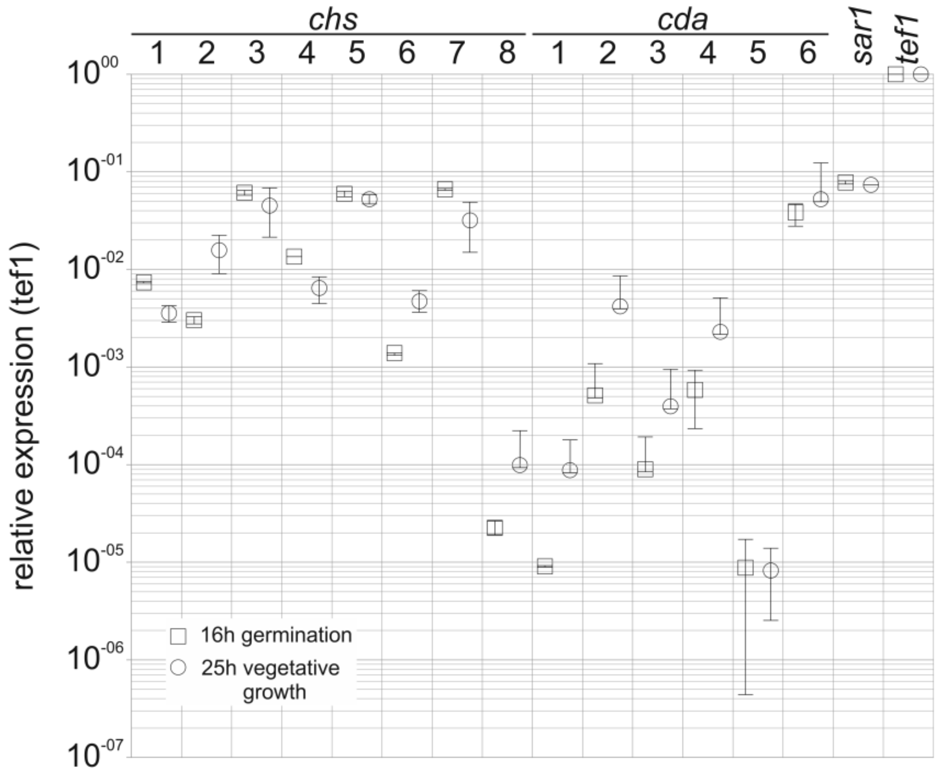

S2 Figure. Relative expression levels of chitin synthase and chitin deacetylase genes.

Expression analysis was performed with all chitin synthase (*chs*) and chitin deacetylase (*cda*) genes by relating transcript levels of *sar1* (housekeeping), *chs1-8* and *cda1-6* to expression of the housekeeping gene *tef1* at 16 h of germination (white squares) or after 24 h of growth on PDA (white circles). Expression of all genes was further normalized to *tef1* using the normalization described by Pfaffl et al., [1]. qRT-PCR data was generated from at least two independent experiments and three technical replicates and analyzed with REST-software. Mean +/- SEM are indicated.
