# Supplementary material for: Chitin and chitosan remodeling defines vegetative development and Trichoderma biocontrol
Source: PLoS Pathog. 2020 Feb 20;16(2):e1008320. doi: 10.1371/journal.ppat.1008320 (PMC7053769; doi:10.1371/journal.ppat.1008320)
Supplement: S3 Fig — (PDF) [file ppat.1008320.s003.pdf]

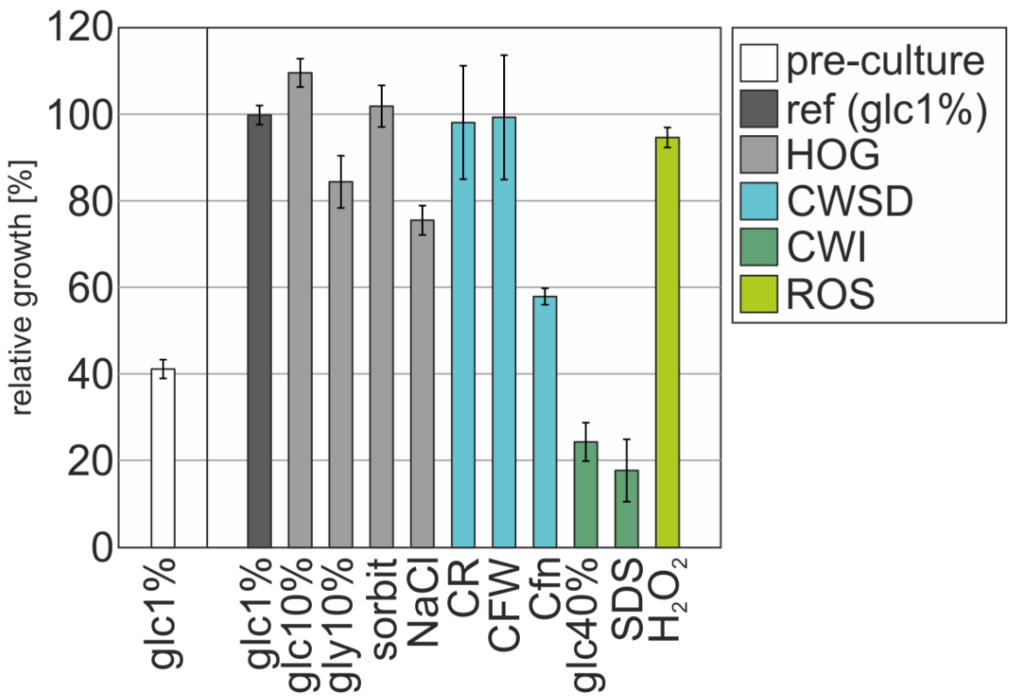

S3 Figure. Biomass formation under stress conditions after 24h.

T. atroviride wild type was grown in liquid minimal medium in a pre-culture for 24 h and then transferred to fresh minimal medium containing one of the indicated stressors: CWI (cell wall integrity disturbing stressors: SDS, 0.025% sodium dodecyl sulfate; glc40 %, 40% glucose; dark green); CWSD (cell wall synthesis disturbing agents, CR, congo red 150 µg/ml; CFW, calcofluor white 200 µg/ml; Cfn, caspofungin 7.5 µg/ml; turquoise); HOG (high osmolarity glycerol, such as 10 % of glucose (glc10%) or glycerol (gly10%); sorbit, 1.2M sorbitol; NaCl, 0.7M NaCl; light grey) and ROS (reactive oxygen species, exposure to H_2O_2, 0.75% H_2O_2; light green). Fresh medium containing only 1 % glucose (glc1%; black) was used as control condition. After 24 h of pre-culture (white bar) and after further incubation in the fresh medium for another 24 h biomass was harvested, dried and weighed and relative growth (colored bars) was inferred by relating the dry weight of each sample to growth on 1 % glucose. Data was generated from at least two independent experiments and two technical replicates. Mean +/- SEM are indicated.
