# Supplementary material for: Chitin and chitosan remodeling defines vegetative development and Trichoderma biocontrol
Source: PLoS Pathog. 2020 Feb 20;16(2):e1008320. doi: 10.1371/journal.ppat.1008320 (PMC7053769; doi:10.1371/journal.ppat.1008320)
Supplement: S4 Fig — (PDF) [file ppat.1008320.s004.pdf]

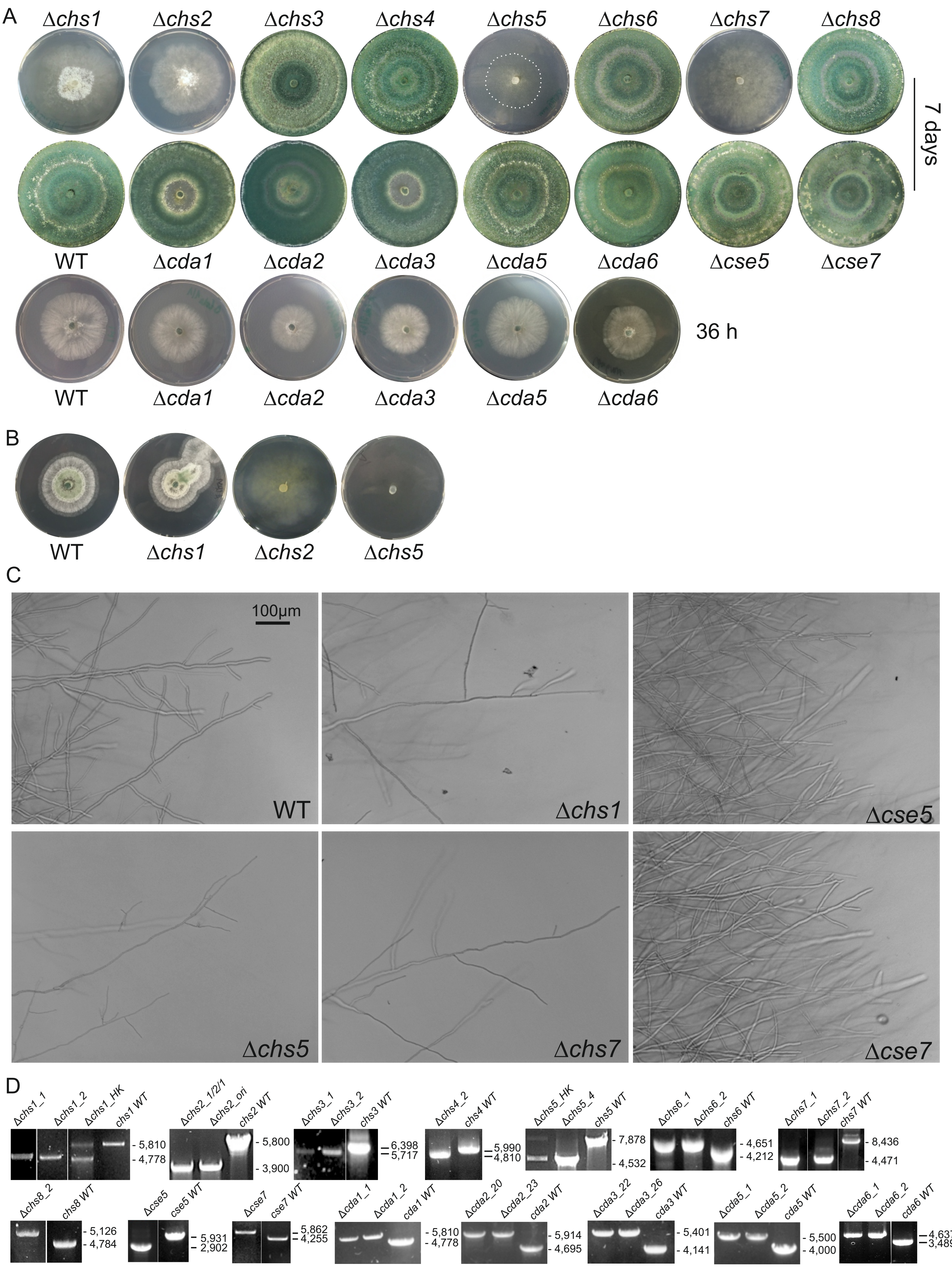
A
Δchs1
Δchs2
Δchs3
Δchs4
Δchs5
Δchs6
Δchs7
Δchs8
7 days
WT
Δcda1
Δcda2
Δcda3
Δcda5
Δcda6
Δcse5
Δcse7
36 h
WT
Δcda1
Δcda2
Δcda3
Δcda5
Δcda6
B
WT
Δchs1
Δchs2
Δchs5
C
100µm
WT
Δchs1
Δcse5
Δchs5
Δchs7
Δcse7
D
Δchs1_1
Δchs1_2
Δchs1_HK
chs1 WT
5,810
4,778
Δchs2_1/2/1
Δchs2_ori
chs2 WT
5,800
3,900
Δchs3_1
Δchs3_2
chs3 WT
6,398
5,717
Δchs4_2
chs4 WT
5,990
4,810
Δchs5_HK
Δchs5_4
chs5 WT
7,878
4,532
Δchs6_1
Δchs6_2
chs6 WT
4,651
4,212
Δchs7_1
Δchs7_2
chs7 WT
8,436
4,471
Δchs8_2
chs8 WT
5,126
4,784
Δcse5
cse5 WT
5,931
2,902
Δcse7
cse7 WT
5,862
4,255
Δcda1_1
Δcda1_2
cda1 WT
5,810
4,778
Δcda2_20
Δcda2_23
cda2 WT
5,914
4,695
Δcda3_22
Δcda3_26
cda3 WT
5,401
4,141
Δcda5_1
Δcda5_2
cda5 WT
5,500
4,000
Δcda6_1
Δcda6_2
cda6 WT
4,637
3,489

S4 Figure. Growth, conidiation and verification of deletion of *chs* and *cda* genes.

(A) Growth on PDA of generated chitin synthase (Δ*chs1-8*) deletion and the exomer adaptor and export chaperon mutants (Δ*cse5, Δcse7*) after 7 days and the chitin deacetylase (Δ*cda1-6*) deletion mutants, and the parental *T. atroviride* strain (WT) after 36h and 7 days. (B) Growth of WT and Δ*chs1,* Δ*chs2* and Δ*chs5* on PDA plates containing 1.2 M sorbitol after 7 days. (C) DIC microscopy of hyphal morphology of *chs*1, *chs*5, *chs*7, *cse*5 and *cse*7 deletion mutants in comparison to the wild type; scale bar = 100 µm. (D) Verification of the integration of the hygromycin B cassette, replacing the respective chitin synthase and chitin deacetylase genes at the correct locus, on 1 % agarose gels. WT amplification served as negative control. Primers used for verification are listed in Table S4, correct height in bp for WT and deletion cassettes in the knock out strains is indicated.
