# Supplementary material for: Chitin and chitosan remodeling defines vegetative development and Trichoderma biocontrol
Source: PLoS Pathog. 2020 Feb 20;16(2):e1008320. doi: 10.1371/journal.ppat.1008320 (PMC7053769; doi:10.1371/journal.ppat.1008320)
Supplement: S5 Fig — (PDF) [file ppat.1008320.s005.pdf]

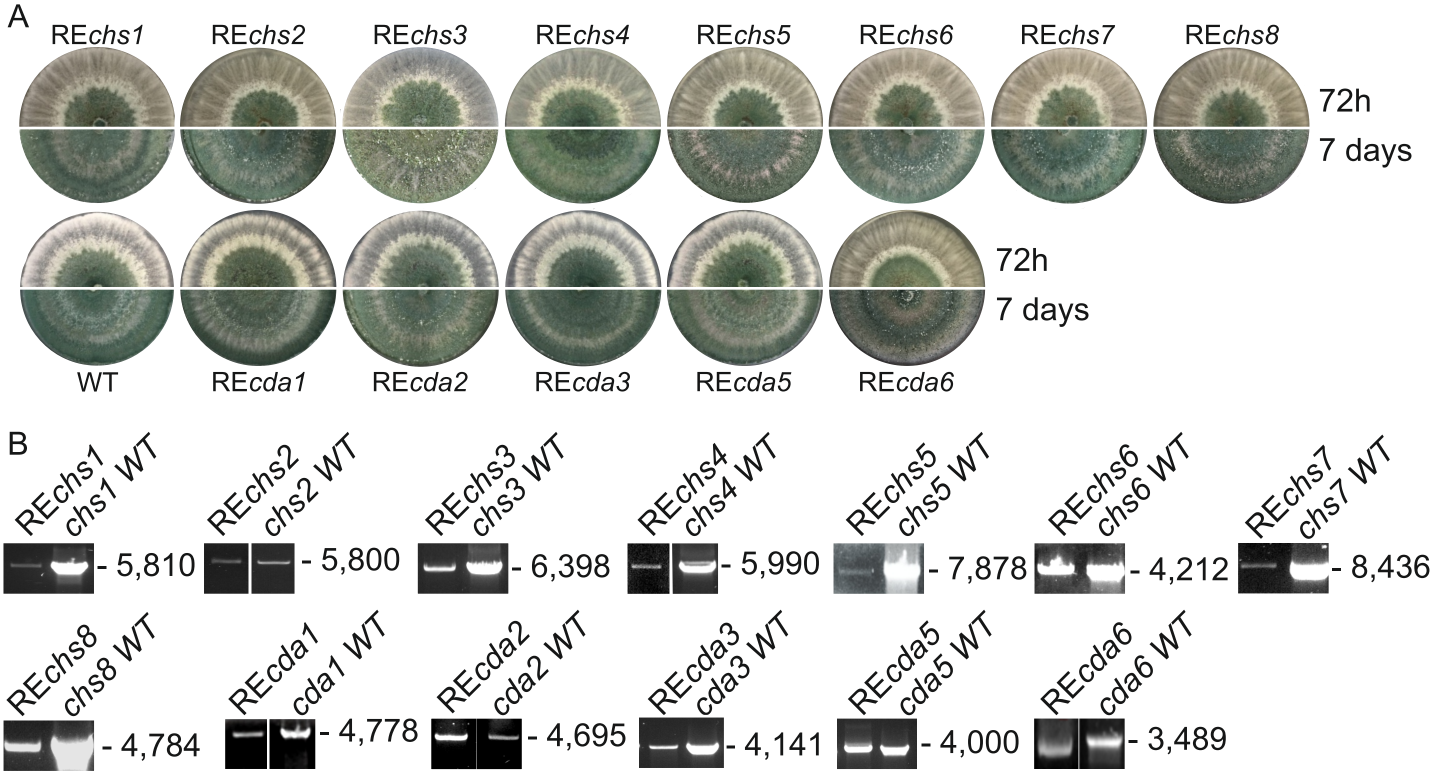

A
REchs1
REchs2
REchs3
REchs4
REchs5
REchs6
REchs7
REchs8
72h
7 days
72h
7 days
WT
REcda1
REcda2
REcda3
REcda5
REcda6
B
REchs1 chs1 WT - 5,810
REchs2 chs2 WT - 5,800
REchs3 chs3 WT - 6,398
REchs4 chs4 WT - 5,990
REchs5 chs5 WT - 7,878
REchs6 chs6 WT - 4,212
REchs7 chs7 WT - 8,436
REchs8 chs8 WT - 4,784
REcda1 cda1 WT - 4,778
REcda2 cda2 WT - 4,695
REcda3 cda3 WT - 4,141
REcda5 cda5 WT - 4,000
REcda6 cda6 WT - 3,489

S5 Figure. Rescue strains.

(A) Growth on PDA of generated rescue strains for chitin synthase (Δ*chs1-8*) deletion and chitin deacetylase (*Δcda1-6*) deletion mutants by complementation with the wild type gene under control of native promoter and terminator elements in comparison to the parental *T. atroviride* strain (WT) after 72h and 7 days on PDA. (B) Verification of the integration of the amdS cassette linked WT gene flanked by native promoter and terminator elements. Primers used for verification are listed in Table S4, correct height in bp is indicated.
