# Supplementary material for: Chitin and chitosan remodeling defines vegetative development and Trichoderma biocontrol
Source: PLoS Pathog. 2020 Feb 20;16(2):e1008320. doi: 10.1371/journal.ppat.1008320 (PMC7053769; doi:10.1371/journal.ppat.1008320)
Supplement: S6 Fig — (PDF) [file ppat.1008320.s006.pdf]

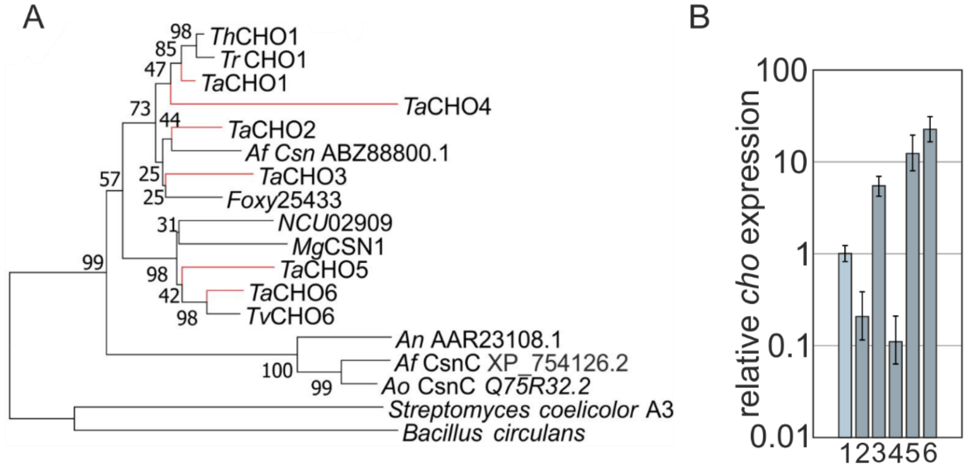

S6 Figure. Phylogenetic analysis of all identified *T. atroviride* chitosanases and their basal transcription levels

(A) Evolutionary tree generated by use of the whole sequence of the indicated enzymes with the phylogenetic analysis software MEGA7, 1,000 bootstraps and the neighbor-joining-method. Numbers next to nodes indicate the bootstrap value. The following enzyme sets were mined from respective databases: Af, *A. fumigatus*; An, *Aspergillus nidulans*; Ao, *Aspergillus oxysporum*; Foxy, *Fusarium oxysporum*; Mg, *Magnaporthae oryzae*; NCU, *Neurospora crassa*; Ta, *T. atroviride*; Th, *T. harzianum*; Tr, *T. reesei*; Tv, *T. virens*. *Streptomyces coelicolor* A3 and *Bacillus circulans* served as outgroup. (B) Basal expression analysis of all six identified chitosanase genes (*cho*1-6) from *T. atroviride* WT by relating transcript levels to expression of *cho1* (light grey bar) after 24 h of growth on PDA. Expression was normalized to *tef1*. qRT-PCR Data was generated from at least two independent experiments and three technical replicates and analyzed with REST-software. Mean +/- SEM are indicated.
