# Supplementary material for: Chitin and chitosan remodeling defines vegetative development and Trichoderma biocontrol
Source: PLoS Pathog. 2020 Feb 20;16(2):e1008320. doi: 10.1371/journal.ppat.1008320 (PMC7053769; doi:10.1371/journal.ppat.1008320)
Supplement: S7 Fig — (PDF) [file ppat.1008320.s007.pdf]

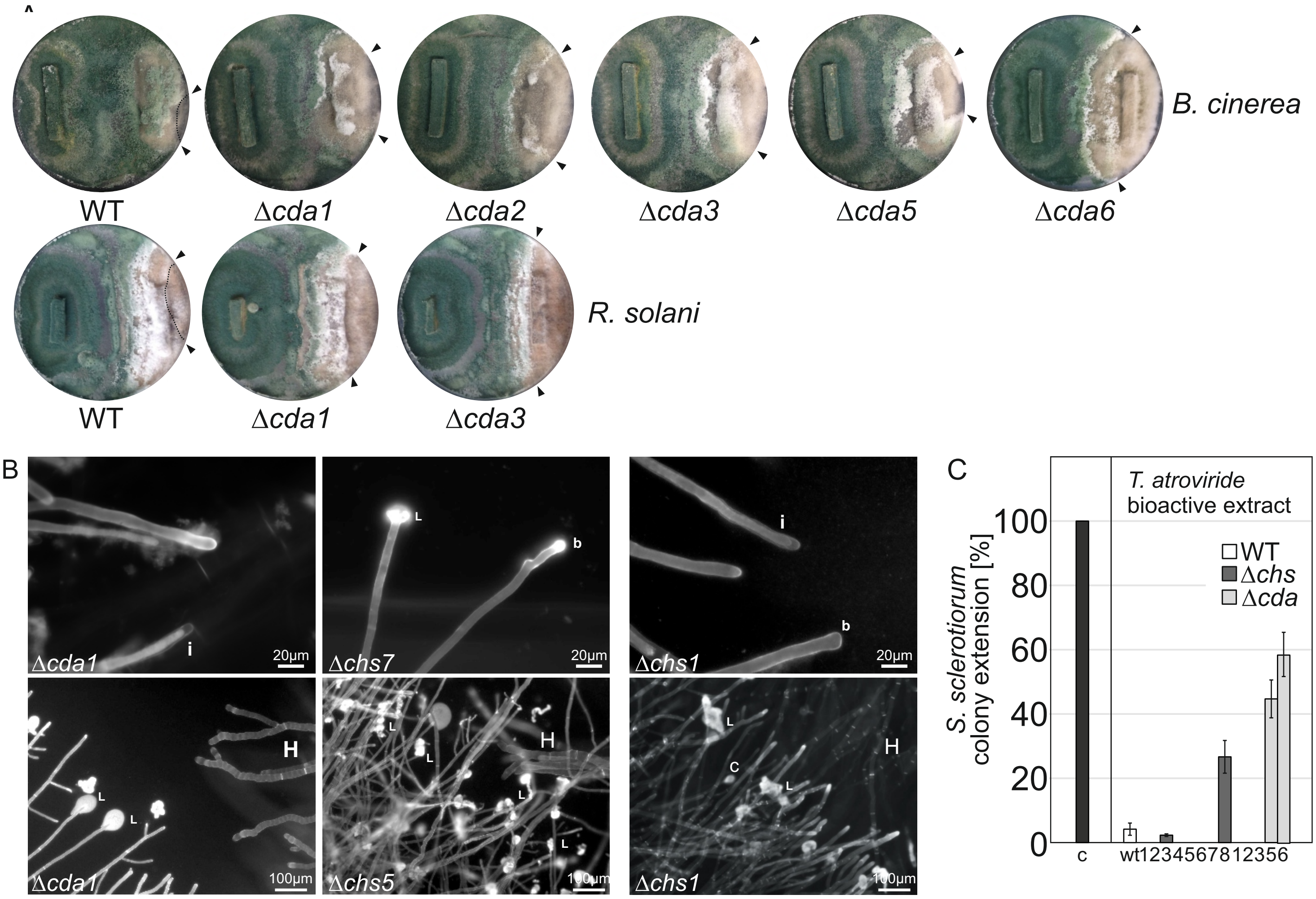
A
B. cinerea
WT
Δcda1
Δcda2
Δcda3
Δcda5
Δcda6
R. solani
WT
Δcda1
Δcda3
B
Δcda1
Δchs7
Δchs1
Δcda1
Δchs5
Δchs1
20µm
100µm
i
b
L
H
C
C
S. sclerotiorum colony extension [%]
T. atroviride bioactive extract
WT
Δchs
Δcda
0
20
40
60
80
100
c
wt12345678 12356

S7 Figure. ***T. atroviride*** **deletion mutants show reduced mycoparasitism on** ***B. cinerea*** **and** ***R. solani*** **while secretion of active compounds is increased.**

(A) Dual confrontation assays against two different hosts: A 5 x 30 mm slice of fully overgrown PDA plates of the indicated *T. atroviride* WT and selected mutant strains was placed on the left side of a PDA plate 5 cm apart from a slice of the hosts *B. cinerea* or *R. solani* and incubated for 5 days. Arrows indicate the zonal overgrowth (dotted line) by *T. atroviride* over the hosts. (B) Microscopic analysis of the confrontation zone of selected mutant strains against *S. sclerotiorum* using CFW staining; L, hyphal leakage; i, intrahyphal growth; c, clamydospores; b, balloon like structure; scale bar indicated. (C) Susceptibility of the phytopathogenic host *S. sclerotiorum* towards secreted antifungal metabolites of *T. atroviride* WT and mutant strains. *Trichoderma* strains (either Wt or Δ) were cultured on plates overlaid with a cellophane disc until ¾ of the plate was overgrown. After removal of the mycelium covered cellophane discs an agar plug of *S. sclerotiorum* was placed upside down on the plates and incubated for 96 h at 25°C in the dark (control, growth of *S. sclerotiorum* on a fresh PDA plate). The inhibition is given in % of each control condition on fresh PDA. Mean +/- SEM of three independent experiments are shown.
