# Supplementary material for: Chitin and chitosan remodeling defines vegetative development and Trichoderma biocontrol
Source: PLoS Pathog. 2020 Feb 20;16(2):e1008320. doi: 10.1371/journal.ppat.1008320 (PMC7053769; doi:10.1371/journal.ppat.1008320)
Supplement: S1 Table — (PDF) [file ppat.1008320.s008.pdf]

S1 Table. CHS4-CSA1 gene cluster.

A	pID *T. atroviride*	pID *T. reesei*	Gene	Predicted function
1	136120	21725	*nag1*	N-acetylglucosaminidase, Chitobiosidase
2		75764		PolyA binding domain
3		57263		Protoporphyrinogen oxidase
4	78499	120618		Thyroid hormone receptor associated
5	311245	75769		Glucose-6-phosphate dehydrogenase
6		120621	*rpl35e*	Ribosomal protein Rpl35 ortholog
7	31030	2826		Chromatin structure and dynamics, SNF2 family DNA-dependent ATPase
8	248552	120623		DNA-binding/SANT; 3 alpha-helices repeats for DNA binding, involved in DNA remodeling
9	226566	57287	*csa1*	ScCHS4 ortholog involved in activation of ScCHS3
10	248556	58188	*chs4*	Class IV chitin synthase orhtolog of ScCHS3
11	226570	2829		Tyrosine kinase
12	320971	2830		Coiled coil protein
13	320974	58058		GTPase/ Guanine nucleotide exchange factor
14	85476	120627		Calcium transporting ATPase
15	301471	58125		Similarity to ScPup3/ beta subunit of the 20S proteasome involved in ubiquitin-dependent catabolism
16	226587	2835		Ubiquitin regulatory protein
17	301471	45912	*rpl36*	Ribosomal protein Rpl36 ortholog
18	301477	120635	*tktA*	Transketolase
19		104786		SNF/SWI
20	148568	104785		Phospho inositide phosphatase
21	85468	75794		Ca^{2+} transcription coactivator/ zink finger
