# Supplementary material for: Chitin and chitosan remodeling defines vegetative development and Trichoderma biocontrol
Source: PLoS Pathog. 2020 Feb 20;16(2):e1008320. doi: 10.1371/journal.ppat.1008320 (PMC7053769; doi:10.1371/journal.ppat.1008320)
Supplement: S2 Table — (PDF) [file ppat.1008320.s009.pdf]

S2 Table. CHS6-CHS5-CHS7 gene cluster.

B	pID *T. atroviride*	pID *T. reesei*	gene	Predicted function
1	298464	63006		Inositol monophosphatase
2		108046		Ribonuclease H
3	216065	71566		Histidine acid phosphatase; pfam00328, Acid phosphate A; Multiple inositol polyphosphate phosphatase
4		122176		Serine/threonine protein kinase
5	142365	122172	*chs5*	Myosin domain CHS
6	290744	122175		Armadillo-type fold, uncharacterized, conserved protein
7	154895	71563	*chs7*	Myosin domain CHS
8	154891	122169		histidine kinase/ hypothetical histidine kinase, part of a two component signal transduction system
9	192857	122168		dehydrogenase kinase, Proteinase inhibitor I9, subtilisin propeptide
10	298475	122166		Candidate cytochrome P450, E-class, group IV, Cytochrome P450 CYP4/CYP19/CYP26 subfamilies;candidate cytochrome P450; Lipid transport and metabolism; homologous to ERG5 C-22 sterol desaturase catalyzes the formation of the C-22(23) double bond in the sterol side chain in ergosterol biosynthesis
11	46717	62975		Rhodanese-related sulfurtransferase; rhodanese homology domain
12	238084	63173	*yip1*	Calcium-binding EF-hand; Yip1 domain-containing protein, four transmembrane alpha helices.
13	262856	42919		(very small) RNA polymerase, I-specific transcription initiation factor rrn7; (Ubiquitin/60s ribosomal protein L40 fusion)
14	298478	71559	*eif-5a*	eIF5A candidate translation initiation factor; homology to the corresponding genes in several filamentous fungi
15	238096	112441	*vps52*	Vacuolar sorting protein VPS52/suppressor of actin Sac2/ ScGARP, (negative regulator of COPII vesicle formation), associated with vacuolar sorting and localization of actin and chitin
16	316418	82531		Predicted GTPase activator protein RabGAP/TBC
17	298483	70795	*eif-5b*	Initiation factor 2; Protein synthesis factor, GTP-binding/ Translation initiation factor 5B (eIF-5B)/ EFTu/EF1A, domain2 Translation elongation factor
18	298484	82534	*hsp88*	Molecular chaperones HSP105/HSP110/SSE1, HSP70 superfamily
19	91144	124228	*chs6*	chitin synthase/hyaluron synthetase
20	238108	82539		Nonsense-mediated mRNA decay 2 protein; MIFG4G-like, type3, Armadillo-type protein binding,
21		82544		Ca2+/H+ antiporter VCX1 and related proteins/Sodium/calcium exchanger membrane region
22	298488	82547		Cys/Met metabolism pyridoxal-phosphate-dependent enzymes/ cystathionine beta-lyase/ pfam01053,
23	142327	124234		hypothetical adenylate cyclase associated protein (CAP)
24	132930	82551		candidate a-mannosyltransferase; Glycosyltransferase Family 62
25	255823	112460		pfam00755, Choline/Carnitine o-acyltransferase, COT/CPT- Carnitine O-acyltransferase CRAT
26	298497	82560		Ribokinase, Carbohydrate transport and metabolism
27	282006	112463		AAA-ATPase, DNA replication licensing factor, MCM3 component
28	298499	82562		Splicing coactivator SRm160/300, subunit SRm300;
29		52839		Cytoskeleton-associated protein and related proteins

30	238142	52841		Cytochrome c heme-binding site; Fungal specific transcription factor activity, zinc ion binding
31	255827	82568		AAA-ATPase; Posttranslational modification, protein turnover
32	216151	70772	*scbst1*	ScBST1, Negative regulator of COPII vesicle formation
