# Supplementary material for: Chitin and chitosan remodeling defines vegetative development and Trichoderma biocontrol
Source: PLoS Pathog. 2020 Feb 20;16(2):e1008320. doi: 10.1371/journal.ppat.1008320 (PMC7053769; doi:10.1371/journal.ppat.1008320)
Supplement: S3 Table — (PDF) [file ppat.1008320.s010.pdf]

S3 Table. Identified promoter response elements.

cellular process	motif	overlap to other known motifs	sequence	abundance CHS 1	CHS 2	CHS 3	CHS 4	CHS 5	CHS 6	CHS 7	CHS 8
**general element**	tata-box		TATAAA	1	0	0	0	0	0	0	2
	CAAT/CAT-box		G[GT]CCAATC[CT]	2	3	3	3	0	2	2	5
**CWI**	CDRE	CRZ1; MET31; MET32; RPN4; STP1	G[ACGT]GGC[GT]CA	7	6	5	8	7	5	5	3
	RLM1		TCTATTTCTAT	3	2	4	0	6	2	1	2
	crzA	MET31; MET32	GTGGCTC	2	1	2	0	4	2	0	3
	Crz1p		[ACGT]GGC[GT]CA	4	4	2	6	6	4	4	3
	CRZ1-1	MET31; RPN4; SNT2; UGA3	[TG]GGCG	3	2	0	5	1	1	7	2
	CRZ1-2		GGGC[GT]	3	2	0	5	1	1	7	2
	CreA	ADR1; MIG1-MIG3; MSN2; MSN4; NHP10; TDA9; YPR022C; ZMS1	[GC][CT]GGGG	4	4	0	3	1	1	2	1
**DEV**	ARE	TEC1	CATTC[CT]	2	1	2	1	2	1	0	2
	AREanalog	ARR1; TEC1	CATTCA	0	0	1	0	2	1	1	0
	AreA/Nit2	DAL80; FZF1; GAT1; GLN3; GZF3	[ACT]GATA[AG]	1	0	0	1	0	0	2	1
	BrlA	MSN2; MSN4; RGM1	[AC][AG]AGGG[AG]	4	2	4	5	2	2	4	3
**HOG**	Sko1p/AtfA-1	CST6; HAC1; SKO1; YAP3	TACGT	2	5	1	0	1	3	2	1

**ROS**	Hap1	STB5; URC2	CGG[ACGT][ACGT][ACGT]TA[ACGT]CGG	2	3	1	5	3	0	7	1
	YAP1	CAD1; YAP7	T[GT]ACTAA	0	0	1	0	0	0	0	0
	AtfA-2	CST6	TGACGTCA	0	2	4	2	2	6	0	4
	CRE-CRZ-Msn2/4	GIS1; MSN2; MSN4; REI1; RGM1; RPH1; YER130C	AGGGG	3	4	8	3	5	0	3	1
**OX stress**	ROX1		[CT][CT][CTA][AC]TTGTTT[TC][TGA][CGA]	2	1	2	3	2	2	1	2
	SKN7		GGC[GC][AGC]	9	9	5	11	1	6	6	8
	STB5		CGGN[GCA][TCA][TCG]A	1	7	1	5	1	1	3	2
	AFT2		[CGT][AG]CACCC[CGT]	0	2	1	0	1	0	0	1
**STRESS general**	TEC1		[AG]CATTC[CT][CT]	2	0	2	1	2	2	0	1
	HSF1		[ACG]T[GA]GAA[CGT][AGT]	7	4	5	5	4	5	3	3
	STP4		[GAT][ACG]TAGCG[CGT][ACG]	0	2	1	3	0	0	5	0

***-enriched motifs**											
**CWI-1**	CHS:2,5,7		acgtgct	1	2	0	0	0	1	2	0
**CWI-2**	CHS:2,5,7		accccct	0	2	0	0	1	0	0	1
**FUSED-1**	CHS:5,7		cccgccgga	0	0	0	0	2	0	1	0
**H2O2-1**	CHS:2,5,6,7,8		gcacgta	0	1	0	0	0	3	1	0
**HOG-1**	CHS:6,8		-								
