# Supplementary material for: Chitin and chitosan remodeling defines vegetative development and Trichoderma biocontrol
Source: PLoS Pathog. 2020 Feb 20;16(2):e1008320. doi: 10.1371/journal.ppat.1008320 (PMC7053769; doi:10.1371/journal.ppat.1008320)
Supplement: S4 Table — (PDF) [file ppat.1008320.s011.pdf]

## S4 Table. Cloning primers.

| Primer Name | name/proteinID (JGI)[a] | Primer sequences for *gene* deletion (5' → 3') |
|---|---|---|
| HY | fwd hygb | TCACGTTGCAAGACCTGCCTGAA |
| YG | rev hygb | TCCGGATGCCTCCGCTCGAAGTA |
| Chs1F1 | 240086/*chs*1 | AATTAGAACTGCGCAGAGAAGCTTGG |
| Chs1F2 | | AGGGCATGAGTTGAGCGCTAACTGAGGGCAGGCCGTACGGTCTCTCAGCGGGCTCTTCCAC |
| Chs1F3 | | GTGGAAGAGCCCGCTGAGAGACCGTACGGCCTGCCCTCAGTTAGCGCTCAACTCATGCCCT |
| Chs1F4 | | CACTCTCCCATTTCCCATTAGTATCACCCTTGCTGCCGATGGGTTATTCACGACAGCC |
| Chs1F5 | | GGCTGTCGTGAATAACCCATCGGCAGCAAGGGTGATACTAATGGGAAATGGGAGAGTG |
| Chs1F6 | | AGATCCACCTTGCCTGCGAGAAGATTATG |
| KO_FwChs1 | | GGCGAGACAGAATTGAGATGGG |
| KO_RvChs1 | | GTACCACGAGATCTGGCTGAAG |
| Chs2F1 | 323101/*chs*2 | AGGCAACGCGTGGACAGGAGGCTT |
| Chs2F2 | | AGGGCATGAGTTGAGCGCTAACTGAGGGCAGGCAGAGGTAGAGGTGATGTGGCGAGCGATGTAGAT |
| Chs2F3 | | ATCTACATCGCTCGCCACATCACCTCTACCTCTGCCTGCCCTCAGTTAGCGCTCAACTCATGCCCT |
| Chs2F4 | | GTCTTTGGCTACTTCGTTAACCTTGTTACCATCTGCTGCCGATGGGTTATTCACGA |
| Chs2F5 | | TCGTGAATAACCCATCGGCAGCAGATGGTAACAAGGTTAACGAAGTAGCCAAAGAC |
| Chs2F6 | | TGGATTTACGGGCTACGCGACGC |
| KO_FwChs2 | | GGTCAAAGGAAAAAATGCCA |
| KO_RvChs2 | | TAGCACCAAGCCGTATCGTT |
| Chs3F1 | 143107/*chs*3 | GACCTAAGTTTGAAGAGCGTCGATGTTAG |
| Chs3F2 | | TGAGTTGAGCGCTAACTGAGGGCAGGCCGAGTGATACAGCGGTTGGTGAGTGAAG |
| Chs3F3 | | CTTCACTCACCAACCGCTGTATCACTCGGCCTGCCCTCAGTTAGCGCTCAACTCA |
| Chs3F4 | | CAAGTCCAAAGTCCAAAGTCCAACTCCATATTCGACAAGACTATCACCGGGCTATT |
| Chs3F5 | | AATAGCCCGGTGATAGTCTTGTCGAATATGGAGTTGGACTTTGGACTTTGGACTTG |
| Chs3F6 | | CAATTCATCTCCCACAGCATCAGAAAC |
| KO_FwChs3 | | GTAGGCTTCTGTCGTATTGGCGAC |
| KO_RvChs3 | | CTTCCAACGGCCTCCTTCAGC |
| Chs4F1 | 248556/*chs*4 | TTTGCACACGCCGCCCGAGAGCACTG |
| Chs4F2 | | AGGGCATGAGTTGAGCGCTAACTGAGGGCAGGCCAACATTCCAAAGGGACAGACAAGTAAAGGCC |
| Chs4F3 | | GGCCTTTACTTGTCTGTCCCTTTGGAATGTTGGCCTGCCCTCAGTTAGCGCTCAACTCATGCCCT |
| Chs4F4 | | CTTGGGCAAAAGGATAGATACAACGGCTGTGCTGCGGAATCATTATCATCTGCTGCC |
| Chs4F5 | | GGCAGCAGATGATAATGATTCCGCAGCACAGCCGTTGTATCTATCCTTTTGCCCAAG |
| Chs4F6 | | CCTGTGGCAAACGACTTCCGCCGA |
| KO_FwChs4 | | GCGGAGTTATTGTTGCGAGAGC |
| KO_RvChs4 | | TTGGCTGTTGGGTATTCGGC |
| Chs5F1 | 142365/*chs*5 | TTCTCTCACTGTTTCCTCATTACTCG |
| Chs5F2 | | AGGGCATGAGTTGAGCGCTAACTGAGGGCAGGCCCGGAGAGCAATGAGGCCAACAGACAGGCG |
| Chs5F3 | | CGCCTGTCTGTTGGCCTCATTGCTCTCCGGGCCTGCCCTCAGTTAGCGCTCAACTCATGCCCT |
| Chs5F4 | | AGTAACGCGTTTCCATTGAGGTCTGCCGATGGGTTATTCACGACAGCCATATTC |
| Chs5F5 | | GAATATGGCTGTCGTGAATAACCCATCGGCAGACCTCAATGGAAACGCGTTACT |
| Chs5F6 | | ACCAGATTAAAACCCTTCCTTCAAGCC |
| KO_FwChs5 | | CTTGTTTATCGCAGCACGTG |
| KO_RvChs5 | | ATTATCAGCGGCAAAGGTCCCT |
| Chs6F1 | 91144/*chs*6 | GATGGGTGTGATGGAGAGCAGTGAC |
| Chs6F2 | | AGGGCATGAGTTGAGCGCTAACTGAGGGCAGGCTCAACCATACAAGTCCAGGCGAACAGCA |
| Chs6F3 | | TGCTGTTCGCCTGGACTTGTATGGTTGAGCCTGCCCTCAGTTAGCGCTCAACTCATGCCCT |
| Chs6F4 | | TGTATTTGTGAGAGATGCAGATGCCAGCTTCTTGTGCTGCGGAATCATTATCATCT |
| Chs6F5 | | AGATGATAATGATTCCGCAGCACAAGAAGCTGGCATCTGCATCTCTCACAAATACA |
| Chs6F6 | | CTTGCGTGGTCGACTGAGGGAC |
| KO_FwChs6 | | ATGGTGGCAGAGAGCGCAGA |
| KO_RvChs6 | | GGGGAACATACCGACGACGG |
| Chs7F1 | 154895/*chs*7 | CGTGGCCGCATAATCAATCG |
| Chs7F2 | | AGGGCATGAGTTGAGCGCTAACTGAGGGCAGGCCACTGCTGAGCGAGGGACAATGGAAACGC |
| Chs7F3 | | TGCTGTTCGCCTGGACTTGTATGGTTGAGCCTGCCCTCAGTTAGCGCTCAACTCATGCCCT |
| Chs7F4 | | TTGTTGCTGCTGTAGTGGTAGGAAATAGTTGAAGTGTGCTGCGGAATCATTATCATCTGCTGCC |
| Chs7F5 | | GGCAGCAGATGATAATGATTCCGCAGCACACTTCAACTATTTCCTACCACTACAGCAGCAACAA |
| Chs7F6 | | TACGATGTTTCTGACGCCGACATCTCC |
| KO_FwChs7 | | GGTGCCAGTGCTTCCCTCTT |
| KO_RvChs7 | | GCTGAGCCGCTCACCGAACGC |
| Chs8F1 | 161127/*chs*8 | AGTAACACAAAGGCGAACCAGCATCATC |
| Chs8F2 | | AGGGCATGAGTTGAGCGCTAACTGAGGGCAGGCTTGAATAGTAGAGAGTTGACGTGAGCGAGTGAA |

| | | |
|---|---|---|
| Chs8F3 | | TTCACTCGCTCACGTCAACTCTCTACTATTCAAGCCTGCCCTCAGTTAGCGCTCAACTCATGCCCT |
| Chs8F4 | | ACAGGAGATGGTCTCTAGCCTCGGTCCCCCATATGTGCTGCGGAATCATTATCATCTGCT |
| Chs8F5 | | AGCAGATGATAATGATTCCGCAGCACATATGGGGGACCGAGGCTAGAGACCATCTCCTGT |
| Chs8F6 | | AAGGCTGTTAGTCGCATTCCTTTTGG |
| KO_FwChs8 | | TGCTAATAGGGAGAGGCGAAGAA |
| KO_RvChs8 | | CGGATGATGGTTTGTGTAAGGACGAG |
| Cda1F1 | 28913/*cda*1 | CGAGAAGCAAGATACTGAGATGAGATGA |
| Cda1F2 | | AGGGCATGAGTTGAGCGCTAACTGAGGGCAGGCTTCGGTAATATCGTCGCCACAAGTGTCACT |
| Cda1F3 | | AGTGACACTTGTGGCGACGATATTACCGAAGCCTGCCCTCAGTTAGCGCTCAACTCATGCCCT |
| Cda1F4 | | CTCCGCCCACAGTTTAAATCCCAATAGTTGCATATTCGACAAGACTATCACCGGGCTATT |
| Cda1F5 | | AATAGCCCGGTGATAGTCTTGTCGAATATGCAACTATTGGGATTTAAACTGTGGGCGGAG |
| Cda1F6 | | AATCTTTTACCCAGCAGTCATTCTATTCATCT |
| KO_Fw Cda1 | | CGACTTCAGCAACCCGTTATCTACTT |
| KO_Rv Cda1 | | TGGAAGAAACTCAGATGGCACATTG |
| Cda2F1 | 147996/*cda*2 | TTGGAGACGGCTATCATGGCTGC |
| Cda2F2 | | AGGGCATGAGTTGAGCGCTAACTGAGGGCAGGCAGGCGGAAGAGGTTGAGGCGTGGCATT |
| Cda2F3 | | AATGCCACGCCTCAACCTCTTCCGCCTGCCTGCCCTCAGTTAGCGCTCAACTCATGCCCT |
| Cda2F4 | | ATTGATTGACGGGCATAGAAGTGTGGTTACATATTCGACAAGACTATCACCGGGCTATT |
| Cda2F5 | | AATAGCCCGGTGATAGTCTTGTCGAATATGTAACCACACTTCTATGCCCGTCAATCAAT |
| Cda2F6 | | TCAAGTGCTGTGTCGTAATGTTTCGG |
| KO_Fw Cda2 | | GCCCATCGACTTCCACATCTACTC |
| KO_Rv Cda2 | | GCCGCAGATGACGCTCCTCCC |
| Cda3F1 | 78914/*cda*3 | GCAACAGCATTTCCCGGGCGAG |
| Cda3F2 | | AGGGCATGAGTTGAGCGCTAACTGAGGGCAGGCCGACAATAGTGCGAGAAAGATGGATAATGA |
| Cda3F3 | | TCATTATCCATCTTTCTCGCACTATTGTCGGCCTGCCCTCAGTTAGCGCTCAACTCATGCCCT |
| Cda3F4 | | AGAACATTGGGCATAAAAGTAGCTGCGTGCATATTCGACAAGACTATCACCGGGCTATT |
| Cda3F5 | | AATAGCCCGGTGATAGTCTTGTCGAATATGCACGCAGCTACTTTTATGCCCAATGTTCT |
| Cda3F6 | | TTCTCAGGCTCATCCAGCACTTTA |
| KO_Fw Cda3 | | TGCCAATCGTCCAAGGTAAAGTCA |
| KO_Rv Cda3 | | GCTTCAGTCCATGTGCAATCCAA |
| CDA4F1 | 291124/*cda*4 | ATGCTCCTTCTTGGGTCGCCTCC |
| Cda4F2 | | AGGGCATGAGTTGAGCGCTAACTGAGGGCAGGCTCTTGGATAATGTTGCCAACGAGTGAATGT |
| Cda4F3 | | ACATTCACTCGTTGGCAACATTATCCAAGAGCCTGCCCTCAGTTAGCGCTCAACTCATGCCCT |
| Cda4F4 | | GTTCGAGCTCGGTGATGACGCCTGTAAATGCAAGCATATTCGACAAGACTATCACCGGGCTATT |
| Cda4F5 | | AATAGCCCGGTGATAGTCTTGTCGAATATGCTTGCATTTACAGGCGTCATCACCGAGCTCGAAC |
| Cda4F6 | | GTTGATAGTCTGGTAGCTGTTGGAT |
| KO_Fw Cda4 | | CTCGGACGCTTTCTTCCAATCTAC |
| KO_Rv Cda4 | | TATCTTCCCTCGGATAACGGA |
| Cda5F1 | 292288/*cda*5 | GAACAATGCAAACAGATACCACGGGAGA |
| Cda5F2 | | AGGGCATGAGTTGAGCGCTAACTGAGGGCAGGCCTATTACGGATTTTCAGGTATTCGACAA |
| Cda5F3 | | TTGTCGAATACCTGAAAATCCGTAATAGGCCTGCCCTCAGTTAGCGCTCAACTCATGCCCT |
| Cda5F4 | | GAATGAGGGTAAAAACAAAATAAATCTAGCATATTCGACAAGACTATCACCGGGCTATT |
| Cda5F5 | | GAATGAGGGTAAAAACAAAATAAATCTAGCATATTCGACAAGACTATCACCGGGCTATT |
| Cda5F6 | | CGACGGGAATCTTGGCGACT |
| KO_Fw Cda5 | | GACCGATTCTCCGACAAGGC |
| KO_Rv Cda5 | | TGCAAACATCGGGCTCCTCTCC |
| Cda6F1 | 142446/*cda*6 | ACACAGTGATAGGAGCTTGGCGAA |
| Cda6F2 | | AGGGCATGAGTTGAGCGCTAACTGAGGGCAGGCGAAAAAACGTCATATCAAAGGTATCTAATG |
| Cda6F3 | | CATTAGATACCTTTGATATGACGTTTTTTCGCCTGCCCTCAGTTAGCGCTCAACTCATGCCCT |
| Cda6F4 | | AAGGGTACCGTTAACTGATCCGAAGTCCGATGCCGATGGGTTATTCACGACAGCCATATTC |
| Cda6F5 | | GAATATGGCTGTCGTGAATAACCCATCGGCATCGGACTTCGGATCAGTTAACGGTACCCTT |
| Cda6F6 | | TGCCCGAGTACAGCGGGCATCCC |
| KO_Fw Cda6 | | GCAACAAGGATCAGGGCAA |
| KO_Rv Cda6 | | CGGTGGTGGCCTTGACGATC |
| Chp1F1 | 179314/*cse*5 | AAGATTGGTCACACGAGGAGAGGCT |
| Chp1F2 | | AGGGCATGAGTTGAGCGCTAACTGAGGGCAGGCGGCCCTAGAGAGTATTTGACAATGGAGATT |
| Chp1F3 | | AATCTCCATTGTCAAATACTCTCTAGGGCCGCCTGCCCTCAGTTAGCGCTCAACTCATGCCCT |
| Chp1F4 | | GGCGATGTTCGTCGTGGCAAGTCCTTGGGACATATTCGACAAGACTATCACCGGGCTATT |
| Chp1F5 | | AATAGCCCGGTGATAGTCTTGTCGAATATGTCCCAAGGACTTGCCACGACGAACATCGCC |
| Chp1F6 | | CTGTTTCCCAGCAAGTCGCACC |
| KO_FW Chp1 | | TTGGCGCCTTTTTCTTTGCT |
| KO_Rv Chp1 | | CCGCTGTGACGTGACCGATG |
| Chp7F1 | 158601/*cse*7 | TCCACTCTCCCATCCCCTCG |
| Chp7F2 | | AGGGCATGAGTTGAGCGCTAACTGAGGGCAGGCGTAATAACAAGAGGCCCAATTCACAAGTC |
| Chp7F3 | | GACTTGTGAATTGGGCCTCTTGTTATTACGCCTGCCCTCAGTTAGCGCTCAACTCATGCCCT |
| Chp7F4 | | ATTAAAGAATCACGCCAACTCAAAGGCCGTCATATTCGACAAGACTATCACCGGGCTATT |
| Chp7F5 | | AATAGCCCGGTGATAGTCTTGTCGAATATGACGGCCTTTGAGTTGGCGTGATTCTTTAAT |
| Chp7F6 | | GGACCTCCCCCCACCATGAT |

| | | |
|---|---|---|
| KO_Fw Chp7 | | AACAGGCCCTCCATATCCCG |
| KO_Rv Chp7 | | TTTCACACTTCGCCTACACCAGCT |

[a]https://genome.jgi.doe.gov/Triat2/Triat2.home.html, Primers used for generation of single gene knock out strains and gene replacement.
