# Supplementary material for: Chitin and chitosan remodeling defines vegetative development and Trichoderma biocontrol
Source: PLoS Pathog. 2020 Feb 20;16(2):e1008320. doi: 10.1371/journal.ppat.1008320 (PMC7053769; doi:10.1371/journal.ppat.1008320)
Supplement: S5 Table — (PDF) [file ppat.1008320.s012.pdf]

S5 Table. qPCR primers.

pID (JGI)[a]	gene	Name	Sequence (5´- 3´)	source
240086	*chs1*	Ta_chs1_qRTs	CGAGGCGAGAGATGGGTTTT	this study
		Ta_chs1_qRTa	GAGTAGACGGCAGCGAAGAA	this study
323101	*chs2*	Ta_chs2_qRTs	GAAGTCTCCGTCTTCAGCGT	this study
		Ta_chs2_qRTa	CAGATCAGCACGACACCAGT	this study
143107	*chs3*	Ta_chs3_qRTs	CCTGGCTCCCTTCAAGGATG	this study
		Ta_chs3_qRTa	ACCAAGAAACGACCAGACCG	this study
248556	*chs4*	Ta_chs4_qRTs	TGTTCTCGTCATCGTCACCG	this study
		Ta_chs4_qRTa	GTTCCAAACGGGGAGAGACA	this study
142365	*chs5*	Ta_chs5_qRTs	ACCAAGAAGGGTGTGAAGCA	this study
		Ta_chs5_qRTa	CCTCAGTGGCGCTGTTGATA	this study
91144	*chs6*	Ta_chs6_qRTs	GTAGAGTCAGGCTGCGGATC	this study
		Ta_chs6_qRTa	CAAACAGGTCTTCGCACTGC	this study
154895	*chs7*	Ta_chs7_qRTs	GGTATCTGCGGTGAGACCAG	this study
		Ta_chs7_qRTa	GGTGAAACATCCGGGCAAAC	this study
161127	*chs8*	Ta_chs8_qRTs	ACTCGGAAGACAGCATCCAC	this study
		Ta_chs8_qRTa	CAATGGTGTATGCCTTGGCG	this study
28913	*cda1*	Ta_cda1_qRTs	AACTGGTTGCGACTCGGAAT	this study
		Ta_cda1_qRTa	GTAGCTGAGAAACGGTCCCC	this study
147996	*cda2*	Ta_cda2_qRTs	ACGCCACCTTCTTCGTCATT	this study
		Ta_cda2_qRTa	CTCCAGCTCGTCATTGCTCA	this study
78914	*cda3*	Ta_cda3_qRTs	CAGAAAGGGCCGCAATTACG	this study
		Ta_cda3_qRTa	TCCGCAATCCAGAGAATCCG	this study
291124	*cda4*	Ta_cda4_qRTs	ACTCACATGAGAACCCAGCG	this study
		Ta_cda4_qRTa	CGACACTCCCTTTTGGTGGT	this study
292288	*cda5*	Ta_cda5_qRTs	TTTGGGCACTCGGAATCACA	this study
		Ta_cda5_qRTa	TGAAAGGTTGGAGGCGGAAA	this study
142446	*cda6*	Ta_cda6_qRTs	GGTACTTGGATGACCTGCCG	this study
		Ta_cda6_qRTa	GCCCGATACATCTGGGTGAA	this study
54365	*cho1*	Ta_cho1_qRTs	GCATTTGGGGTGACACCAAC	this study
		Ta_cho1_qRTa	TTATCGCCGCTCAGACCATC	this study
80199	*cho2*	Ta_cho2_qRTs	GCATTGATGACTTGGACGCC	this study
		Ta_cho2_qRTa	CAGCCATGACACTCAAGGGT	this study
145108	*cho3*	Ta_cho3_qRTs	CGATACCAACACAGAGCCGA	this study
		Ta_cho3_qRTa	CGTCGCCAAGGAAACCAATG	this study
197013	*cho4*	Ta_cho4_qRTs	GGAGAGGCTTTCCTGGCATT	this study
		Ta_cho4_qRTa	CAGGCTGGTAACGGGTTGAT	this study
216890	*cho5*	Ta_cho5_qRTs	CGTGACCTTTGAGCCAGAGT	this study
		Ta_cho5_qRTa	GAGAGCATCAGAGACCTCGC	this study
16857	*cho6*	Ta_cho6_qRTs	CTGGGGAGATACCAATGGCG	this study
		Ta_cho6_qRTa	ATGCCGTTACCAAAGCAAGC	this study
154960	*crh1a*	Ta_crh1a_qRTs	GGCTACACCGATTTCTCCAA	this study
		Ta_crh1a_qRTa	CTCGACCTTGATGCTCTGC	this study
311401	*crh1b*	Ta_crh1b_qRTs	GCACGCCTGATAGCTCCCCG	this study
		Ta_crh1b_qRTa	CACAACCATCAGCAGCGACG	this study
299218	*crh2*	Ta_crh2_qRTs	CGTCTGCCAAGGGTTCCGC	this study
		Ta_crh2_qRTa	TGTTGCTACCACTGTCGCTGC	this study
136120	*nag1*	Ta_nag1_qRTs	ACTGTCGTTCAATCGTGGCT	this study
		Ta_nag1_qRTa	GTTGAAAGAGTCGCCGTTGG	this study
131598	*chi18-5*	Ta_chi18-5_qRTs	TGGATCTGGAAGCTGGGAGA	this study
		Ta_chi18-5_qRTa	AGCTGTAGTATGCCTGTGCG	this study
302419	*prb1*	Ta_prb1_qRTs	TCCAACACTGCTACCAACACAATC	this study
		Ta_prb1_qRTa	TTTCACTTCATCCTTCGCTCCA	[2]

300828	*tef1*	Ta_tef1_qRTs	GTGCGGTGGTATTGACAAGC	this study
		Ta_tef1_qRTa	ACTTCCAGAGGGCAATGTCG	this study
301975	*sar1*	Ta_sar1_qRTs	GCCGTTTCTGAGGATGAGCT	this study
		Ta_sar1_qRTa	CTGATACCGTCACCGTAGCC	this study

[a]https://genome.jgi.doe.gov/Triat2/Triat2.home.html, qRT-PCR primers used in this study.
