# Supplementary material for: Chitin and chitosan remodeling defines vegetative development and Trichoderma biocontrol
Source: PLoS Pathog. 2020 Feb 20;16(2):e1008320. doi: 10.1371/journal.ppat.1008320 (PMC7053769; doi:10.1371/journal.ppat.1008320)
Supplement: S6 Table — (PDF) [file ppat.1008320.s013.pdf]

S6 Table. Generated knockout and rescue strains.

knockout strain	pID (JGI)[a]	genotype[b]	rescue strain[c]	genotype
Δchs1	240086	*Δchs1::hphB*	***REchs1***	*Δchs1::hphB, pchs1-chs1-tchs1:amdS*
Δchs2	323101	*Δchs2::hphB*	***REchs2***	*Δchs2::hphB, pchs2-chs2-tchs2:amdS*
Δchs3	143107	*Δchs3::hphB*	***REchs3***	*Δchs3::hphB, pchs3-chs3-tchs3:amdS*
Δchs4	248556	*Δchs4::hphB*	***REchs4***	*Δchs4::hphB, pchs4-chs4-tchs4:amdS*
Δchs5	142365	*Δchs5::hphB*	***REchs5***	*Δchs5::hphB, pchs5-chs5-tchs5:amdS*
Δchs6	91144	*Δchs6::hphB*	***REchs6***	*Δchs6::hphB, pchs6-chs6-tchs6:amdS*
Δchs7	154895	*Δchs7::hphB*	***REchs7***	*Δchs7::hphB, pchs7-chs7-tchs7:amdS*
Δchs8	161127	*Δchs8::hphB*	***REchs8***	*Δchs8::hphB, pchs8-chs8-tchs8:amdS*
Δcda1	28913	*Δcda1::hphB*	***REcda1***	*Δcda1::hphB, pcda1-cda1-tcda1:amdS*
Δcda2	147996	*Δcda2::hphB*	***REcda2***	*Δcda2::hphB, pcda2-cda2-tcda2:amdS*
Δcda3	78914	*Δcda3::hphB*	***REcda3***	*Δcda3::hphB, pcda3-cda3-tcda3:amdS*
Δcda5	292288	*Δcda5::hphB*	***REcda5***	*Δcda5::hphB, pcda5-cda5-tcda5:amdS*
Δcda6	142446	*Δcda6::hphB*	***REcda6***	*Δcda6::hphB, pcda6-cda6-tcda6:amdS*
Δcse5	179314	*Δcse5::hphB*	***REcse5***	*Δcse5::hphB, pcse5-cse5-tcse5:amdS*
Δcse7	158601	*Δcse7::hphB*	***REcse7***	*Δcse7::hphB, pcse7-cse7-tcse7:amdS*

[a]https://genome.jgi.doe.gov/Triat2/Triat2.home.html
[b]knockout strains created in this study by gene replacement with the hygromycin B resistance cassette
[c]rescue strains created in this study by re-introduction of the WT gene under control of native promoter and terminator elements linked to a acetamidase gene from A. fumigatus in the respective knockout strains
